# Perception of effective access to health services in Territorial Spaces for Training and Reincorporation, one year after the peace accords in Colombia: a cross-sectional study

**DOI:** 10.12688/f1000research.21375.2

**Published:** 2020-06-09

**Authors:** Julián Alfredo Fernández-Niño, Lud Magdy Chavarro, Ana Beatriz Vásquez-Rodríguez, Maylen Liseth Rojas-Botero, Ginna Esmeralda Hernández-Neuta, Ana Maria Peñuela-Poveda, David Alejandro Rodríguez

**Affiliations:** 1 Department of Public Health, Universidad del Norte, Barranquilla, Colombia; 2 Migration and Health programme, International Organization for Migration, Bogotá, Colombia; 3 Facultad Nacional de Salud Pública, Universidad de Antioquia, Medellín, Colombia; 4 Ministerio de Salud y Protección Social, Bogotá, Colombia

**Keywords:** Health Services Accessibility, Armed Conflicts, Colombia

## Abstract

**Backgrounds**: The signing of the peace accords in Colombia created challenges that are inherent to post-conflict transitions. One of those is the process of reintegrating ex-combatants into society, in which ensuring their rights to health is a particularly significant challenge in rural areas affected by armed conflict. These areas, known as Territorial Spaces for Training and ReintegrationReincorporation (ETCR, in Spanish), are geographically dispersed throughout 24 municipalities and 13 departments in Colombia. This study aimed to describe how ex-combatants in ETCR regions perceived access to health services one year after the signing of the peace accords.

**Methods:** A descriptive, cross-sectional study was performed between September and October 2018. It included 591 adults and their families, from 23 ETCRs. The study was designed, culturally validated, and piloted. Interviewers were trained and a structured survey was administered containing five dimensions that characterized the perception of effective access to health services.

**Results**: The majority of interviewees were women, heads of household, young adults, ex-combatants, and residents in an ETCR. In total of 96.4% were enrolled in Colombia’s subsidized health system, and 20.8% indicated that a member of their household required emergency health services. The regional health center provided the majority of the services. Most of those surveyed (96.0%) reported that they did not have to pay for the services, and that they received respectful (91.6%) and good quality (66.6%) care. There were few referrals to disease prevention and health promotion activities, and only 19.0% of households reported having been visited by extramural health care teams, whose activities were highly valued (80%). Lastly, there was little knowledge about community health activities.

**Conclusions**: While residents of ETCR regions have a favorable perception of their access to health services, they need to be made aware of extramural and public health activities.

## Introduction

As a human right, health is crucial to human and social development. Societies that reach an optimal level of health are able to ensure that their populations attain higher individual and collective levels of human development ^1, 2^. For a society that is going through a period of post-conflict, guaranteeing health as a right demonstrates the State’s interest in taking steps towards renewing the social bonds among the parties that were broken by the conflict. Effective access to health services expresses the recognition of the right to health, making it possible to prevent illness and to address the population’s health needs ^3^.

Historically, the concept of *access to* health services has had multiple definitions that correspond to the structural values of each health system. Most of the models and definitions of access are based on the logic of markets and the satisfaction of needs, with very few taking the perspective of health as a right ^4^. United Nations General Observation 14 defines the effective exercise of the right to health as availability, accessibility, acceptability, and quality —elements or dimensions that are essential to enjoying effective access to health services ^2^.

Providing access to health services for the entire population is an enormous challenge for a society that finds itself in the midst of armed conflict or in a period of post-conflict. This is the case of Colombia after the signing of the Final Accord (November 2016) between the federal government and FARC-EP (Fuerzas Armadas Revolucionarias de Colombia—Ejército del Pueblo; Revolutionary Armed Forces of Colombia—People’s Army) revolutionary forces to End the Armed Conflict and Build a Stable and Lasting Peace ^5^ ( https://www.cancilleria.gov.co/sites/default/files/Fotos2016/12.11_1.2016nuevoacuerdofinal.pdf 5). As a result, Colombia finds itself navigating through a critical process that has great social, economic, and political impact. A key factor that ensures the end of the conflict and a stable and lasting peace is preparing for the reincorporation of ex-combatants into all areas of civilian life —economic, political, and social. To this end, Territorial Spaces for Training and Reincorporation (ETCR, in Spanish) were established to relocate former FARC-EP members and establish the means and inputs needed to fulfill the promises and responsibilities that were set out in the accord, including supplying this population with comprehensive humanitarian aid through national and international organizations ^6^.

One promise that is aimed at ensuring the reincorporation of ex-combatants is promoting and guaranteeing their right to health in rural areas and small towns that lack the institutional and budgetary capacities to provide social and health services, and that historically have not received State investments. The Colombian government established eight dimensions for the path of reincorporation, one of which is access to the General Social Security Health System (SGSSS, in Spanish). It also established health services as a fundamental right to be guaranteed along with returning these people and their families to legality. Colombia is a good example of the comprehensiveness that these programs need when it comes to ex-combatants who are on the path to reincorporation, in the short-term. Meanwhile, long-term and sustainable reincorporation into civilian life will be ensured by including crucial elements such as effective health care and ongoing access to health services through enrollment in the health system. This will contribute to individual well-being, continued legality, and a solid ground on which to build stable and lasting peace.

Some of the projects were developed with the above challenges in mind and in accordance with the reference framework on which the path was laid out, such as Health for Peace, led by the Ministry of Health and implemented by three United Nations agencies (UNFPA, PAHO/WHO, and IOM). This project was created in order to strengthen institutional and community capacities to address health needs in the rural areas where the ETCRs are located, through already-established regional health centers as well as local primary hospitals. Health services were also complemented by the development of extramural strategies and community surveillance, led by regional health professionals with the support of local public health leaders who were trained by SENA as part of the project.

In this way, the “Health for Peace” contributes to the implementation of Point 1 in the Implementation Framework Plan (IFP) of the Final Accord, which relates to comprehensive rural reform and is associated with the implementation of Territorial Health Plans in the priority municipalities. It also complemented the emergency response by the Colombian government during the first year after the accord, which consisted of a physician, a nursing professional, and an ambulance for each ETCR. These health workers held positions that at first lacked the conditions needed to guarantee the quality of health care, given that the infrastructure and human skills for providing health services in the regions where the ETCRs are located has historically been insufficient, and non-existent in some cases. This emergency measure was gradually phased out as the Health for Peace project was being implemented. In this sense, the main objective of the Project was to strengthen the local capacity for improving access to comprehensive Primary Health Care services —with an emphasis on sexual and reproductive rights, mental health, preventing consumption of psychoactive substances, and children’s health and malnutrition— thereby addressing the needs that had previously been identified by the United Nations Verification Mission in the ETCR. Furthermore, in order to promote access to comprehensive health services, support was provided to implement an extramural strategy as part of Primary Health Care, as well as to develop a strategy for community participation and surveillance. This proposal can be considered to be the specific development of strengthening health services in the rural area through recent technical developments by the Ministry of Health, such as the National Public Health Plan, but adapted to the context of ex-combatants, their families, and the residents of these regions, with the advantage of receiving technical and financial support from agencies in the United Nations system.

Health care interventions by governments and international agencies are challenged by budgetary limitations due to the economic crisis, as well as by operational limitations in light of the large number of ETCRs and their geographic dispersion throughout Colombia. Added to this is the increased demand for health services, especially by pregnant women and young children, as well as signs of a lack of confidence in the quality of health care and its capacity to respond to needs, all of which could affect the perception of access to health services in the ETCRs.

Based on this approach, perception serves as a differentiating construct. It is defined as “ *a mental process that enables forming judgments or categories that define reality, of interest to health because it enables identifying the population’s impressions of health status, the resolving of needs, and the interventions performed.”* ^7^ Perception more accurately shows “ *the appreciation of reality by obtaining a set of concepts and attitudes that are associated with the health care provided and received.”* ^8^ This is an alternative to representing access to health services based solely on the indicators that are generated by health care providers and authorities, which explore comprehensive access management in a simpler manner. Some examples of this can be found in most of the current surveys that have been developed in Colombia, including the Evaluation of Services by Health Promoter Entities (EPS in Spanish) ^9^, as well as the National Health and Nutrition Survey in Mexico (ENSANUT, in Spanish) ^10^. The objective of the study herein was to describe how ex-combatants living in the ETCRs perceived access to health services one year after the signing of the peace accords. This study captured the specific impressions of effective access to health services by taking into account the health needs of the intervention community, which can be accomplished by measuring two dimensions that make up part of the perception of a community: contact coverage and satisfaction ^11^. From the perspective of the right to health as an analytical framework, these two dimensions come closest to actual access to services, and can best represent that.

To this end, a survey was developed and administered to the population residing in the ETCRs in order to explore the perception of access to services from the perspective of guaranteeing the right to health.

## Methods

### Study design and population

A cross-sectional, descriptive study was performed. The target population was ex-combatants and their families who were living in 23 Territorial Spaces for Training and Reincorporation (ETCR, in Spanish) in September 2018. In each ETCR, a characterization from July 2018 of ex-combatants and their families was used as a sampling framework, which was developed by the IOM (International Organization for Migration) field team. The family was the study unit, although the informants were the heads of the households or family members who had the most information about the state of health of the family members, which in most cases were women. According to inclusion criteria, at least one of the family members was a FARC-EP ex-combatant, demobilized after the signing of the Final Accord, residents of an ETCR, who consented to participate. There were not any exclusion criteria.

### Sample

A stratified sampling was calculated with a finite population, taking each ETCR as a non-proportional stratum, and based on the following parameters: total census population of 3,792 people, grouped into 848 families as of the date of this study; 2) expected prevalence of poor perception of effective access of 40%; 3) 95% confidence interval; 4) 23 strata; and 5) design effect of 2. This resulted in a minimum sample size of 529 people, with 23 participants needed from each ETCR. With this sample, estimators for the total population can be calculated by taking into account the source of variance by cluster of total residents in each ETCR, but it does not permit calculating separate estimates for each ETCR.

The expected value of 40% for effective access is a conservative estimate given that in the few studies conducted in population without payment capacity in Colombia, the perception of low effective access found is greater than 50% ^12^. However, the sample size needed to be adjusted slightly in order to include parameters having more extreme expected values, such as use of emergency services (20%) and health insurance (80%). The first value derives from the same reports where the emergency services have usually the worst evaluation. On the other hand, given that the commitment of the Peace process was to guarantee universal coverage for all ex-combatants, we considered that a value of 80% for health insurance is conservative considering that it had been a year after the signing of the agreement.

In this way, the sample would enable obtaining a precise estimation of the majority of the parameters of interest. Given the small number of people living in some of the ETCRs, it was decided that all the families would be included when clusters (ETCR) had less than 25 people, and a minimum of 30 people would be included when clusters had over 25 people. This projection, which was slightly larger than the sample needed, was made in order to compensate for losses in the ETCRs that had less than the required number of families, and to anticipate non-differential losses in information.

The use of a simple random sampling had been planned for selecting the subjects within each ETCR. However, the ex-combatants considered this to be inconvenient, and therefore, a house-to-house interview (convenience sampling) was conducted in order to identify the sample for this investigation. All the houses located in the ETCR were inhabited by ex-combatants of the FARC-EP and their families. The interviewers applied the survey in all the homes where the head of the household was present and met the inclusion criteria simply following the order of the first houses they found in each ETCR until they completed the required sample size. Results did not present a clear bias, given the high degree of homogeneity in the expected living conditions of the subjects, the high coverage of the sample (which in five cases included the entire population), and the fact that the interviewers did not apply any arbitrary criteria for choosing the interviewees, nor was there any self-selection. This was the best possible approach given the conditions of the population that was required for the sample. The representatives of each ETRC did not accept the possibility of random sampling.

Additionally, it is important to note that little information exists about the health status of this population, and the construction of a sampling framework was not very feasible because of protection considerations.

### Data collection instruments and procedures

A survey was designed in order to identify *perception of effective access to health services* in the ETCRs.

No structured instruments exist for measuring effective access to health services for ex-combatants in a context such as Colombia. Therefore, a measuring instrument was designed based on a literature review of perception and effective access to health services from the perspective of the right to health. A review was also conducted of the instruments that are currently available on this topic, particularly those used in rural areas. Based on the findings, *perception of effective access to health services* was defined as the instrument’s main construct, which was structured according to the domains or dimensions needed for reflecting that construct from the perspective of health as a right, that is: *availability, accessibility, acceptability, quality, contact coverage, and satisfaction.*

For the sociodemographic, emergency services, and promotion and prevention modules, some of the items in the instruments related to access to health services for Colombian households were adapted. This included questions from the Survey of Access to Health Services for Colombian Households (EASS) developed by Arrivillaga, Aristizábal, Pérez & Estrada ^13^ and the instrument that is used to evaluate the services provided by the EPS ^9^. These were adapted in order to ensure traceability with the majority of the indicators that are available for the rest of the Colombian population. The questions that were aimed at determining perception, extramural activities, and community monitoring were designed based on the literature review and the objectives and developments of the Health for Peace project, which served as references. (see glossary of terms in the supplementary material).

The final instrument contained 65 questions that were divided into three modules: 1) sociodemographics of the household (7 questions); 2) perception of effective access to primary health care services (54 questions), which was divided into insurance (2 questions), emergency services (19 questions), health promotion and disease prevention programs (23 questions), and extramural services (10 questions); and 3) community health monitoring (4 questions). All the questions were posed to the head of the family or a member of the household. The head of the family was the person that the family members recognized as most able to provide information about the participants. The study instrument and an English translation are available as extended data ^14, 15^.

The survey was revised and validated using a technique that was applied by professionals from the IOM and MSPS. Face and content validation was performed; the experts evaluated relevance and comprehensiveness of the items in two focal groups reviewing each item of the instrument. Besides, the interviewers used a socio-cultural method to adapt the terms and definitions in the survey for better comprehension on the part of the population of interviewees. The interviewers were nursing professionals located in each municipality and were highly experienced in community work and in collecting information for public health. The team’s researchers trained the interviewers in administering the surveys during a one-day session, which ended with a qualitative evaluation.

A pilot test was conducted, which consisted of administering the survey in six ETCRs, with a total of 43 interviews. Based on this, the first socio-cultural validation of the survey was performed, in which the interviewers had the opportunity to provide feedback based on their experience administering the survey. A second socio-cultural validation was conducted during an in-person training session with the interviewers from each municipality. This involved reviewing each dimension, its acceptability, and the interviewees’ understanding. In this session, role-playing and simulation workshops were conducted and the entire instrument and its application were reviewed.

For the final administration of the survey, an interviewer was assigned to each one of the 23 ETCRs. These ETRCs were located in the following municipalities: Anorí, Dabeiba, Ituango, Remedios and Vigia del Fuerte (Department of Antioquia); Arauquita (Arauca); La Montañita and San Vicente (Caquetá); Buenos Aires, Miranda, and Caldono (Cauca); “Region between La Paz and Manaure” (Cesar); “Region between Carmen de Darien y Riosucio” (Chocó); Fonseca (La Guajira); San José del Guaviare (department of Guaviare); Vista, Mesetas and La Macarena (Meta), Tumaco (Nariño), Tibú (Norte de Santander), Puerto Asis (Putumayo), Icononzo and Planadas (Tolima).

The survey was conducted in the homes of ex-combatants, and each interviewer interviewed approximately 25 to 30 families between September 2018 and October 2018, thereby obtaining the sample that had been calculated previously. When the interviewers administered the survey to the informants, they were asked if they had gone to receive care from one of the services evaluated within 4 weeks prior to the survey, to avoid memory bias and to ensure that the project’s ongoing activities would be evaluated. One of the team’s researchers continually supervised the collection of information in order to ensure the quality of the data.

### Statistical analysis

A basic descriptive analysis was performed, in which the qualitative variables were described with proportions and the quantitative variables were described with measures of central tendency and dispersion. In all cases we worked with the valid percentage. All of the analyses were performed with SPSS © version 23.

### Ethical considerations

This research was approved by the Ethics Committee of the Health Division of the Universidad del Norte ( *Committee Minutes* # 198).

The objectives of the study were explained to all the interviewees and oral informed consent was obtained before the survey was administered. The informed consent was oral because we wanted to protect the identity of the ex-combatants and it was preferable not to have any document signed by them with personal information for security reasons as well as to make feel them confident. In order to obtain the oral informed consent, after explaining the objectives of the study, and guaranteeing confidentiality, each person was asked if he/she understood the explanation, if not, the explanation was repeated, and if he/she said yes, it was recorded in the questionnaire whether or not the person agreed to participate in the survey. The answer to this question is recorded in each questionnaire. All these procedures were approved by the Ethics Committee.

We guarantee that all respondents were informed of the objectives and freely invited to participate. They were also told that no benefit or detriment would result from their decision about whether or not to participate.

## Results

A total of 591 people were interviewed, with a 100% response rate. They were asked about their sociodemographic characteristics, enrollment in the health system, and health services used by themselves or by a family member. The majority of the interviewees were women, heads of household, young adults, did not identify with a cultural group or ethnicity, and were ex-combatants residing in the 23 Territorial Spaces for Training and Reincorporation (ETCR) ( Table 1).

**Table 1.** Social and demographic characteristics of the participants in the perception of effective access to health services survey, in the Territorial Spaces for Training and Reincorporation (ETCR, in Spanish), according to sex.

	Men	Women	Total
**Age**			
Mean (SD)	35.6 (10.4)	31.3 (8.4)	33.0 (9.5)
Median (Rq)	36.0 (15,0)	30.0 (11.0)	32 (12.3)
Min – Max	18 – 71	16 – 62	16 – 71
Coefficient of variation (CV%)	29.2%	26.8%	28.8%
Total	236	345	582
**Place of residence**			
Cabecera municipal (urban) % (n)	4.9 (11)	1.3 (4)	2.7 (15)
Other (rural settlements) % (n)	62.7 (141)	74.1 (237)	69.4 (379)
Other (dispersed rural) % (n)	32.4 (73)	24.7 (79)	27.8 (152)
Total (n)	225	320	546
**Number of people in household**			
Mean (SD)	2.3 (1.4)	2.9 (1.3)	2.7 (1.4)
Median (Rq)	2.0 (2.0)	3.0 (1.0)	2.0 (1.0)
Min – Max	1 – 8	1 – 10	1 – 10
CV%	60.9%	44.8%	51.9%
Total	235	340	576
**Ethnicity**			
Indigenous % (n)	16.4 (39)	16.7 (57)	16.6 (96)
Raizal of the archipelago % (n)	0.4 (1)	0.6 (2)	0.5 (3)
Black, Multiracial, Afro-Colombian, African Descent % (n)	16.4 (39)	4.7 (16)	9.5 (55)
Romani (ROM) % (n)	0.0 (0)	0.0 (0)	0.0 (0)
Palenquero de San Basilio % (n)	0.0 (0)	0.0 (0)	0.0 (0)
None (multiracial) % (n)	58 (138)	62.5 (213)	60.7 (352)
White % (n)	7.6 (18)	13.8 (47)	11.2 (65)
Other % (n)	1.3 (3)	1.8 (6)	1.6 (9)
Total (n)	238	341	580
Group *			
Victims % (n)	10.5 (25)	10.5 (36)	10.4 (61)
Disabled % (n)	7.5 (18)	3.2 (11)	5.0 (29)
Ex-combatants % (n)	75.3 (180)	69.2 (238)	71.7 (419)
Displaced % (n)	1.7 (4)	9.6 (33)	6.3 (37)
None % (n)	4.6 (11)	5.5 (19)	5.1 (30)
Other % (n)	0.4 (1)	2 (7)	1.4 (8)
Total (n)	239	344	584
**Type of housing**			
Housing % (n)	36.8 (84)	41.3 (140)	39.6 (225)
Rented room % (n)	58.4 (133)	54.91 (186)	56.2 (319)
Other type of housing (carp, tent, car, boat, natural shelter, etc) % (n)	4.8 (11)	3.8 (13)	4.2 (24)
Total (n)	228	339	568
**Role in the family**			
Head of household % (n)	87.8 (209)	49 (169)	64.7 (378)
Husband/Wife / Partner % (n)	8.8 (21)	48.4 (167)	32.4 (189)
Son/daughter % (n)	2.5 (6)	0.9 (3)	1.5 (9)
Other % (n)	0.8 (2)	1.7 (6)	1.4 (8)
Total (n)	238	345	584
**Highest education level**			
None % (n)	3 (7)	2.9 (10)	2.9 (17)
Incomplete elementary % (n)	25.7 (61)	21.4 (74)	23.2 (135)
Complete elementary % (n)	17.3 (41)	20.3 (70)	19.2 (112)
Secondary (until 9th grade) % (n)	9.3 (22)	13.3 (46)	11.7 (68)
Complete high school (until 11 ^th^ grade ) % (n)	19 (45)	15.1 (52)	16.6 (97)
Incomplete high school % (n)	25.7 (61)	27 (93)	26.4 (154)
Total (n)	237	345	583

*Groups defined as mutually exclusive categories, based on self-identification.

The majority said that they were enrolled in the subsidized General Social Security Health System. Only 3.6% (21 people) said they were not enrolled in the health system, either because they did not apply, or they applied but were not registered by the system.

With regard to emergency services, 20.8% said that they or a member of their household needed those services for some type of health condition or illness the month prior to the survey. Of those, 16.4% did not seek medical care. The majority of those who did seek care went to the health centers or hospitals in their local area, primarily because they were close to their homes. Obstetric care and illnesses that are prevalent in children were the most reported reasons why people sought medical attention ( Table 2).

**Table 2.** Access to emergency services by participants in the perception of effective access to health survey in the Territorial Spaces for Training and Reincorporation (ETCR, in Spanish). IPS (in Spanish) - Service provider institutions, EPS (in Spanish) - Health promoter entities.

	Region					Total
	Andina	Amazonía	Caribe	Orinoquía	Pacífico	
**During the four weeks before taking this survey, did** **you or a member of your household require emergency** **services for a condition or illness?**						
Yes % (n)	20.9 (39)	9.6 (11)	19.7 (12)	16.1 (14)	19.1 (26)	17.4 (102)
Yes, but did not get medical attention % (n)	4.8 (9)	1.7 (2)	0.0 (0)	1.1 (1)	5.9 (8)	3.4 (20)
No % (n)	73.3 (137)	88.7 (102)	80.3 (49)	82.8 (72)	75 (102)	78.8 (462)
Don’t know % (n)	1.1 (2)	0.0 (0)	0.0 (0)	0.0 (0)	0.0 (0)	0.3 (2)
Total (n)	187	115	61	87	136	586
**If you received medical attention, where did you or the** **member of your household go for your condition or** **illness?**						
IPS (local health center or hospital) % (n)	86.8 (33)	72.7 (8)	83.3 (10)	64.3 (9)	61.5 (16)	75.2 (76)
IPS (health center or hospital at the closest town or city % (n)	7.9 (3)	27.3 (3)	16.7 (2)	28.6 (4)	26.9 (7)	18.8 (19)
Pharmacy % (n)	2.6 (1)	0.0 (0)	0.0 (0)	0.0 (0)	3.8 (1)	2.0 (2)
Other % (n)	2.6 (1)	0.0 (0)	0.0 (0)	7.1 (1)	7.7 (2)	4.0 (4)
Total (n)	38	11	12	14	26	101
**What is the main reason why you or your family member** **decided to go there?**						
Like how they treat patients % (n)	2.8 (1)	0.0 (0)	8.3 (1)	14.3 (2)	3.8 (1)	5.1 (5)
It was covered by health plan % (n)	16.7 (6)	36.4 (4)	58.3 (7)	7.1 (1)	30.8 (8)	26.3 (26)
Know and trust the physician/healer there % (n)	27.8 (10)	0.0 (0)	0.0 (0)	14.3 (2)	0.0 (0)	12.1 (12)
They have better service (staff. supplies and medications) % (n)	2.8 (1)	9.1 (1)	0.0 (0)	0.0 (0)	3.8 (1)	3.0 (3)
The service is quicker than at the local IPS (health center or hospital) % (n)	11.1 (4)	0.0 (0)	16.7 (2)	7.1 (1)	7.7 (2)	9.1 (9)
It is closer to home % (n)	33.3 (12)	45.5 (5)	16.7 (2)	57.1 (8)	23.1 (6)	33.3 (33)
It is inexpensive/ does not cost anything % (n)	0.0 (0)	0.0 (0)	0.0 (0)	0.0 (0)	7.7 (2)	2.0 (2)
Not affiliated with any health system % (n)	0.0 (0)	0.0 (0)	0.0 (0)	0.0 (0)	3.8 (1)	1.0 (1)
Other % (n)	5.6 (2)	9.1 (1)	0.0 (0)	0.0 (0)	19.2 (5)	8.1 (8)
Total (n)	36	11	12	14	26	99
**What was the main reason why you or a member of your** **household sought emergency care?**						
Obstetrics care % (n)	30.6 (11)	18.2 (2)	16.7 (2)	14.3 (2)	15.4 (4)	21.2 (21)
Common childhood illness % (n)	22.2 (8)	0.0 (0)	41.7 (5)	14.3 (2)	11.5 (3)	18.2 (18)
Malnutrition and/or nutritional deficiency % (n)	0.0 (0)	0.0 (0)	0.0 (0)	7.1 (1)	0.0 (0)	1.0 (1)
Infectious disease % (n)	2.8 (1)	9.1 (1)	16.7 (2)	14.3 (2)	19.2 (5)	11.1 (11)
Chronic illness (diabetes, high blood pressure, gastritis, obesity, headache, migraine) % (n)	2.8 (1)	27.3 (3)	0.0 (0)	21.4 (3)	11.5 (3)	10.1 (10)
Musculoskeletal disease % (n)	8.3 (3)	9.1 (1)	16.7 (2)	0.0 (0)	3.8 (1)	7.1 (7)
Skin problems % (n)	0.0 (0)	18.2 (2)	0.0 (0)	0.0 (0)	0.0 (0)	2.0 (2)
Other % (n)	33.3 (12)	18.2 (2)	8.3 (1)	28.6 (4)	38.5 (10)	29.3 (29)
Total (n)	36	26	26	14	26	99
**How much time past between the moment you arrived** **at the emergency room and when you were seen by a** **physician?**						
I was seen immediately % (n)	31.6 (12)	27.3 (3)	41.7 (5)	78.6 (11)	20.0 (5)	36.0 (36)
In 30 minutes % (n)	39.5 (15)	9.1 (1)	25 (3)	7.1 (1)	36.0 (9)	29.0 (29)
Between 31 minutes and 1 hour % (n)	13.2 (5)	18.2 (2)	8.3 (1)	0.0 (0)	16.0 (4)	12.0 (12)
Between 1 and 2 hours % (n)	7.9 (3)	9.1 (1)	0.0 (0)	7.1 (1)	16.0 (4)	9.0 (9)
Over 2 hours % (n)	7.9 (3)	36.4 (4)	25 (3)	7.1 (1)	8.0 (2)	13.0 (13)
Don’t know % (n)	0.0 (0)	0.0 (0)	0.0 (0)	0.0 (0)	4.0 (1)	1.0 (1)
Total (n)	38	11	12	14	25	100
**Do you feel the time you or the member of your** **household waited to be seen was:**						
Very long % (n)	10.5 (4)	45.5 (5)	8.3 (1)	14.3 (2)	30.8 (8)	19.8 (20)
Long % (n)	5.3 (2)	9.1 (1)	8.3 (1)	0.0 (0)	7.7 (2)	5.9 (6)
Average % (n)	23.7 (9)	18.2 (2)	41.7 (5)	7.1 (1)	15.4 (4)	20.8 (21)
Short % (n)	60.5 (23)	27.3 (3)	41.7 (5)	78.6 (11)	42.3 (11)	52.5 (53)
Don’t know % (n)	0.0 (0)	0.0 (0)	0.0 (0)	0.0 (0)	3.8 (1)	1.0 (1)
Total (n)	38	11	12	14	26	101
**Did the emergency room physician clearly say what** **health condition you or your household member had?**						
Yes % (n)	83.8 (31)	70 (7)	75 (9)	78.6 (11)	64 (16)	76.3 (74)
No % (n)	13.5 (5)	30 (3)	25 (3)	21.4 (3)	28 (7)	21.6 (21)
Don’t know % (n)	2.7 (1)	0.0 (0)	0.0 (0)	0.0 (0)	4 (1)	2.1 (2)
Total (n)	37	10	12	14	25	97
**With regard to the recommendations or care for your** **health condition, besides medication, did you receive any** **from the emergency room physician?**						
The physician explained them and I understood % (n)	56.8 (21)	72.7 (8)	75 (9)	76.9 (10)	50 (12)	61.9 (60)
The physician explained them and I understood only some % (n)	35.1 (13)	9.1 (1)	0.0 (0)	15.4 (2)	29.2 (7)	23.7 (23)
The physician explained them and I did not understand any % (n)	0.0 (0)	9.1 (1)	0.0 (0)	0.0 (0)	0.0 (0)	1 (1)
The physician did not give explanations % (n)	8.1 (3)	9.1 (1)	25 (3)	7.7 (1)	20.8 (5)	13.4 (13)
Total (n)	37	11	12	13	24	97
**With regard to the medications that were prescribed, did** **the person who gave you the prescription:**						
Give explanations and did you understand all of them % (n)	76.5 (26)	62.5 (5)	72.7 (8)	92.9 (13)	48 (12)	69.6 (64)
Give explanations and did you understand only some of them% (n)	23.5 (8)	0.0 (0)	18.2 (2)	0.0 (0)	24 (6)	17.4 (16)
Give explanations and did you not understand any of them% (n)	0.0 (0)	12.5 (1)	0.0 (0)	0.0 (0)	0.0 (0)	1.1 (1)
Did not give explanations % (n)	0.0 (0)	12.5 (1)	9.1 (1)	7.1 (1)	24 (6)	9.8 (9)
Don’t know % (n)	0.0 (0)	12.5 (1)	0.0 (0)	0.0 (0)	4 (1)	2.2 (2)
Total (n)	34	8	11	14	25	92
**Were you able to get all of the medications?**						
Yes. all of them % (n)	88.2 (30)	55.6 (5)	100 (10)	64.3 (9)	60 (15)	75.0 (69)
No % (n)	5.9 (2)	22.2 (2)	0.0 (0)	35.7 (5)	20 (5)	15.2 (14)
Only some % (n)	5.9 (2)	22.2 (2)	0.0 (0)	0.0 (0)	16 (4)	8.7 (8)
Don’t know % (n)	0.0 (0)	0.0 (0)	0.0 (0)	0.0 (0)	4 (1)	1.1 (1)
Total (n)	34	9	2	14	25	92
**Where did you get the medications?**						
In the pharmacy at the local IPS (health center or hospital) where we went for emergency care % (n)	62.1 (18)	14.3 (1)	9.1 (1)	41.7 (5)	20.8 (5)	36.1 (30)
At a place that was run by the EPS, outside the IPS % (n)	13.8 (4)	14.3 (1)	45.5 (5)	0.0 (0)	33.3 (8)	21.7 (18)
A private pharmacy % (n)	24.1 (7)	42.9 (3)	36.4 (4)	58.3 (7)	45.8 (11)	38.6 (32)
Other % (n)	0.0 (0)	28.6 (2)	9.1 (1)	0.0 (0)	0.0 (0)	3.6 (3)
Total (n)	29	7	11	12	24	83
**What was the main reason why you could not get the** **medications?**						
The EPS where I am enrolled did not cover the medication and I did not have money to buy it % (n)	10 (1)	0.0 (0)	0.0 (0)	11.1 (1)	18.2 (2)	9.5 (4)
The IPS did not have the medication % (n)	40 (4)	25 (2)	25 (1)	33.3 (3)	18.2 (2)	28.6 (12)
The place where I had to go to get the medication was far from the IPS where I was treated % (n)	0.0 (0)	0.0 (0)	25 (1)	0.0 (0)	18.2 (2)	7.1 (3)
They did not explain how to get them % (n)	0.0 (0)	25 (2)	0.0 (0)	11.1 (1)	0.0 (0)	7.1 (3)
Other % (n)	50 (5)	37.5 (3)	50 (2)	44.4 (4)	27.3 (3)	40.5 (17)
Don’t know % (n)	0.0 (0)	12.5 (1)	0.0 (0)	0.0 (0)	18.2 (2)	7.1 (3)
Total (n)	10	8	4	9	11	42
**Was the care that you or your household member were** **given respectful and in accordance with your culture and** **gender, and was your opinion as a user of the service** **taken into account?**						
Yes % (n)	94.4 (34)	90.9 (10)	91.7 (11)	100 (14)	81.8 (18)	91.6 (87)
No % (n)	5.6 (2)	9.1 (1)	8.3 (1)	0.0 (0)	13.6 (3)	7.4 (7)
Don’t know % (n)	0.0 (0)	0.0 (0)	0.0 (0)	0.0 (0)	4.5 (1)	1.1 (1)
Total (n)	36	11	12	14	22	95
**If you had an opportunity to choose, would you return to** **the local IPS emergency room (health center or hospital)** **to be treated again?**						
Yes % (n)	91.2 (31)	63.6 (7)	91.7 (11)	92.9 (13)	52.4 (11)	79.3 (73)
No % (n)	5.9 (2)	36.4 (4)	8.3 (1)	7.1 (1)	47.6 (10)	19.6 (18)
Don’t know % (n)	2.9 (1)	0.0 (0)	0.0 (0)	0.0 (0)	0.0 (0)	1.1 (1)
Total (n)	34	11	12	14	21	92
**In general, do you consider the quality of the emergency** **services to have been**						
Very good % (n)	20 (6)	0.0 (0)	0.0 (0)	9.1 (1)	4.3 (1)	9.5 (8)
Good % (n)	53.3 (16)	33.3 (3)	81.8 (9)	81.8 (9)	47.8 (11)	57.1 (48)
Average % (n)	16.7 (5)	44.4 (4)	9.1 (1)	9.1 (1)	30.4 (7)	21.4 (18)
Poor % (n)	3.3 (1)	11.1 (1)	0.0 (0)	0.0 (0)	13 (3)	6 (5)
Very poor % (n)	3.3 (1)	11.1 (1)	0.0 (0)	0.0 (0)	4.3 (1)	3.6 (3)
Don’t know % (n)	3.3 (1)	0.0 (0)	9.1 (1)	0.0 (0)	0.0 (0)	2.4 (2)
Total (n)	30	9	11	11	23	84

More than 50% reported that the waiting time to receive care was short, the majority said they did not have to pay for the services (96.0%), that they had been treated respectfully (91.6%), that the quality of care was good (66.6%), and that they had received all the medications that they needed (75.0%) ( Table 2).

In contrast, 25% said that the waiting time was long or very long (over 2 hours for 13.0% of cases), 21.6% did not receive a diagnosis from the professional who provided the service, and 23.9% could not obtain some or any of the medications that were indicated. The reasons mentioned for not being able to obtain the medications were: they were not available at the IPS (service provider institutions) pharmacy or the pharmacy was not open at night or on the weekend, the place where they are obtained is far from where they received the health services, and the medications were not covered by their benefits plan ( Table 2).

**Figure 1.**
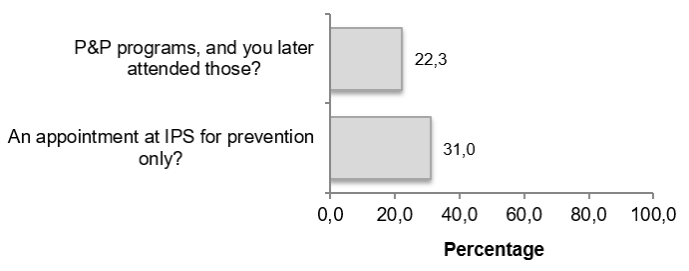
Over the four weeks before taking this survey, were you or someone in your household offered:

The low number of referrals to prevention and health promotion activities was notable, especially in the case of children under 1 year of age (11.9%) (69) and pregnant women (8.1%) (48) ( Figure 1– Figure 3). Nonetheless, this may be due to the time at which the report was requested, which in this case was four weeks before the survey was administered. Of those who indicated that they used promotion and prevention services, the majority (53.4%) said that the appointment was made quickly (the next day) and the majority (68%) perceived the quality of the service to be good, including recommendations, care, and exams that were always appropriate for their age. Most of those surveyed (86.8%) also said that they would definitely return to use the services that these types of programs offered ( Figure 4 and Figure 5).

**Figure 2.**
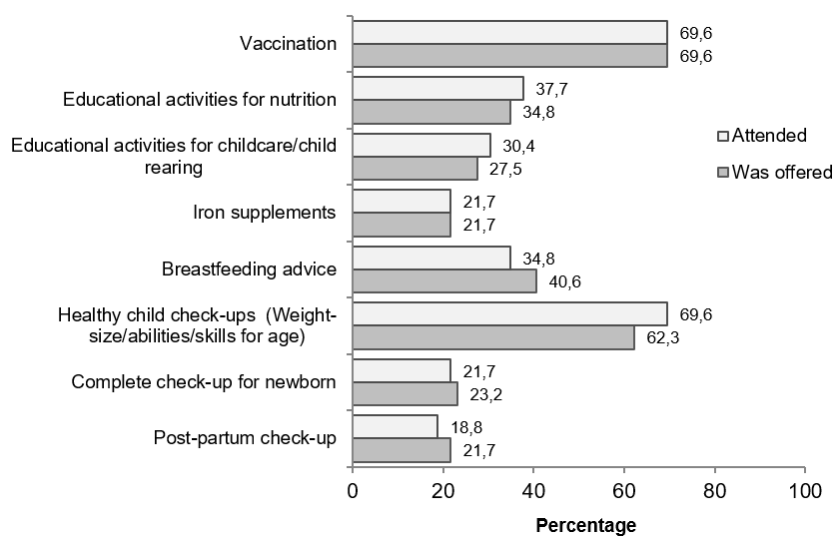
A total of 11.7% (69) of households had at least one child under 1 year of age. They were asked about whether they were offered and attended health promotion and disease prevention activities and programs over the four weeks before the survey was taken.

**Figure 3.**
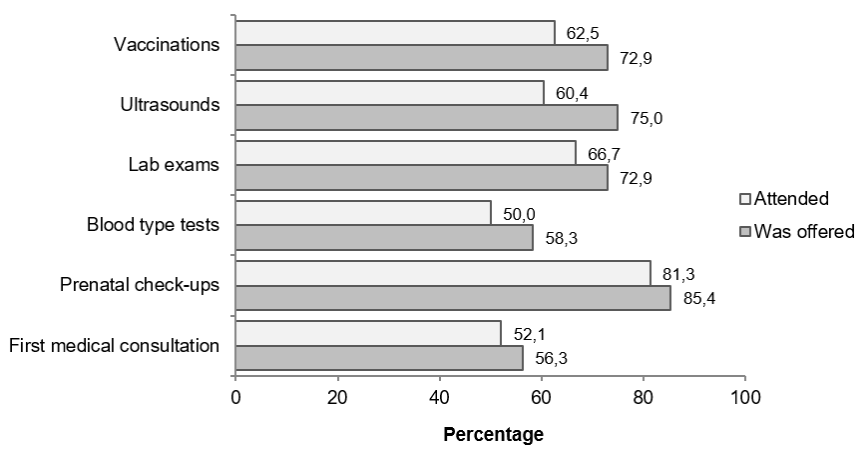
A total of 8.1% (48) of the households reported at least one pregnant woman. They were asked about whether they were offered and attended health promotion and disease prevention activities and programs over the four weeks before the survey was taken.

**Figure 4.**
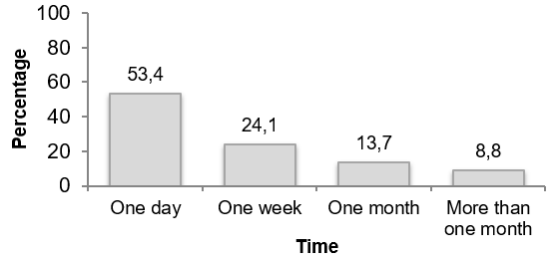
Opportunity to get an appointment with promotion and prevention programs (n= 249).

**Figure 5.**
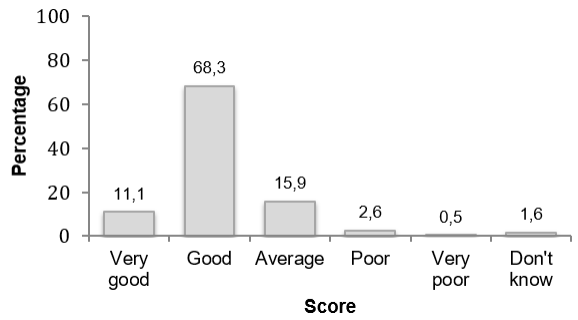
Perception of the quality of the service provided by promotion and prevention programs (n=189).

**Figure 6.**
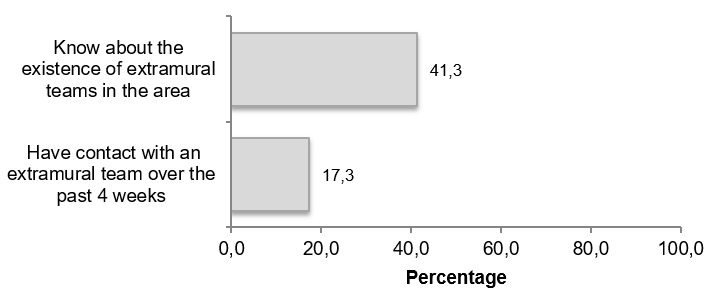
Did you or someone in your household: (n=591).

**Figure 7.**
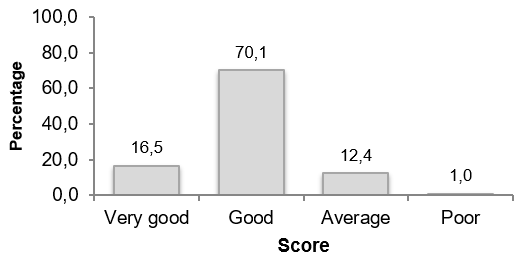
Perception of the quality of the extramural care in the area (n=97).

A total of 17.3% of households received visits from extramural health teams. Over 80.0% of participants positively evaluated those activities in terms of the relevance of the health education they received, how they were treated, the opportunity to receive new services, and the quality of the services ( Figure 6 and Figure 7).

**Figure 8.**
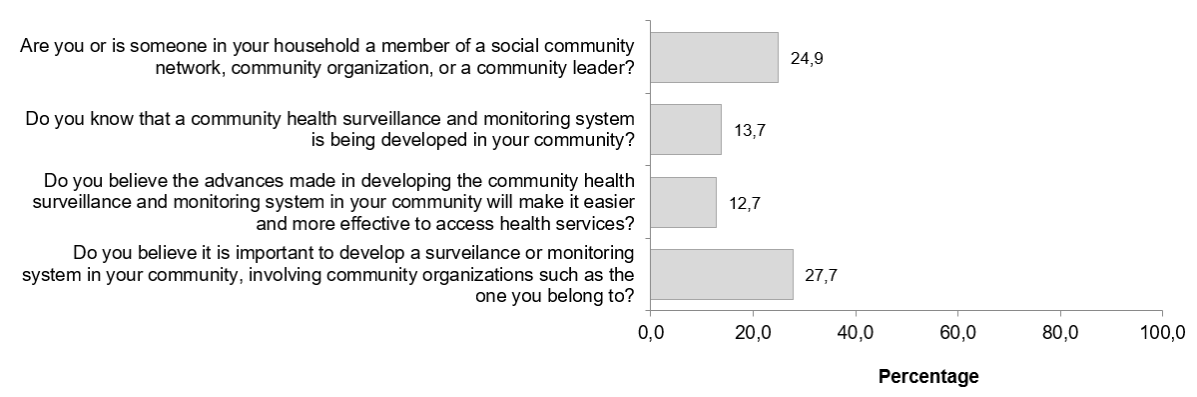
Community surveillance (n=591).

Lastly, participation in community health activities and knowledge about them were low. In all, 13.7% knew about the development of a community health monitoring system, and 27.7% considered the development of those types of spaces to be important, where different community organizations get involved ( Figure 8).

## Discussion

Fride (cited by Márquez, 2013 and Molano, 2015) defined *post-conflict* as “ *the period in which past hostilities have decreased to the level that is needed for beginning reincorporation and rehabilitation activities”* ^16, 17^. The signing of the accord not only involved the political will of institutions but also a commitment by all sectors in the society to building a stable and lasting peace ^5, 17^.

Processes to disarm, demobilize, and reintegrate (DDR) ex-combatants into civilian life, and their return to legality, are crucial challenges for ensuring a lasting peace in the post-conflict period. Negative repercussions have resulted from inadequately planning these processes or from their absence, such as in the case of Namibia ^18^. These consequences not only affect the quality of life of former combatants, including stigmatization and unemployment, but they also put at risk the strengthening of democratic institutions and the social and economic stability of countries, triggering a reactivation of violence ^18^. The inclusion of these dimensions in Colombia´s peace process is noteworthy, when compared to other successful national policies aimed at the social and economic reincorporation of illegally armed people and groups. For example, while reincorporation polices in Indonesia and the Congo have been successful and innovative, and reconciliation mechanisms have been comprehensive and involved the community, health care was mentioned only as a condition that needed to be met, or it was limited to health days or the delivery of medical kits ^18^.

DDR processes are indispensable to building peace. Not only do they contribute to ensuring security after conflict by reducing the number of people who return to armed conflict, but more importantly, these programs can make a significant impact and are socially important in terms of capacity-building, local governance, economic projects, economic development conditions, and the reconstruction and reconciliation needed in countries that have undergone these types of internal conflicts ^19^.

While prioritizing the right to health for ex-combatants represents important progress in public policy, Colombia’s health system faces challenges that need to be studied in depth along with academic efforts ^20, 21^. This issue is important in two ways. First, because of the dispersion of the locations where these populations reside. This concerns point number 1 in the accord (comprehensive rural reform and the development of the National Rural Health Plan), which for some experts means that the health system needs to expand primary health care and change its tendency towards institutionalization and medicalization, since that tendency has led to the intramural provision of services, with little or no access in rural areas to health services, medications, diagnostic services, and/or treatment ^22, 23^. And second, because health is indispensable to the path of reincorporation. Ensuring it provides the conditions needed for the reincorporation process and guarantees its success.

In this study, after conducting the literature review on access to health services, we decided to administer a survey in order to estimate the perception of effective access to health services among ex-combatants located in ETCRs in rural areas of Colombia. Obtaining an estimation based on perception provides a clearer picture of how these populations have experienced health care services when seeking those services or being treated, and whether that experience of contact with health services has resulted in actually realizing that they can access the services, be treated, and resolve their health needs. This is what resoundingly represents actual access to health services and the actual quality of those services.

With regard to the instrument, the survey was designed with this interpretative framework of perception so as to directly evaluate access to health services for ex-combatants within the framework of legality. Given that this type of information is not available from other countries that have implemented post-conflict reincorporation processes, the results from the present work provide a new methodology and information that can be used to identify the health status of populations that became invisible because of their situation as active actors in armed conflict. The present work also offers an objective evaluation of progress on one of the dimensions of the reincorporation program. This has previously been studied only in terms of percentage of people enrolled in the health system, which does not reflect access to health services for ex-combatants in the rural areas where they are located.

In terms of the sociodemographic composition and enrollment in the health system, the results very closely reflect the global population that was in the process of reincorporation in the ETCRs as of the date the survey was administered. Proportional similarities were found when comparing our data with official 2018 data from the Agency for and Normalization (ARN) ^24^, with the same trends continuing until the last report by the agency in March 2019 ^25^. This suggests that the estimations obtained in the study herein adequately represent the characteristics and perceptions of access to health services in the general population of ex-combatants residing in these spaces. With regard to the composition of the population, the majority (56.43%) in the ARN report were 26 to 40 years of age, and the majority (nearly 80%) of those surveyed by the study herein fell within this same age range. With regard to the highest education level, ARN reported that incomplete or complete elementary school was most frequently (32.29%) reported by the population of ex-combatants, while our results found that the largest percentage of this population reported attending high school, when considering elementary, incomplete high school, and complete high school. For sex, the percentage distributions also differed, which could be explained by who responded to the survey. The unit of observation in the study herein was the household and the majority of the respondents were women, whereas the ARN study surveyed individuals in the process of reintegration, the majority of which were men.

With regard to enrollment in the health system, ARN reported that 80% of the ex-combatants in the process of reintegration were enrolled in the system in September 2018, the majority of whom belonged to the subsidized system. And the work herein shows that over 90% belonged to this system. It is interesting to note that the 10% that was not enrolled in the system had not applied, suggesting that health services had high administrative capacities for this population, and that the full orientation and consulting process provided by the path to reincorporation was functioning for the ex-combatants in the program. This was also seen in the 2014 survey of perceptions by ARN, in which 82% of those who said they received support to access the health system reported that this strategy helped them to gain access ^26^. However, it is important to note these are not precise comparisons since these data, which come from ARN´s programs, include ex-combatants who demobilized before the peace agreement, the majority of which moved to urban areas. These results are similar to the findings from the 2015 National Demographics and Health Survey (ENDS, in Spanish) of the rural population, which reported 94% enrollment, the majority in the subsidized system ^27^.

It is difficult to compare the results from our survey because of a lack of historical data on the health status of populations belonging to armed groups and their access to health services. This fact also makes these results highly valuable as a baseline for planning the provision of health services for this new population that is joining the system.

For the purpose of comparing the results from our survey on the health status of the ex-combatant population, we compared our findings with access to health services by the general rural population, which currently may be the best control population given the rural location of the ETCRs. The sociodemographic composition can be compared with the general rural population by using the results from the 2015 ENDS survey ^28^, which contains very precise measurements of the population by location. Similarities with our estimates can be seen, in which the overall characteristics of these populations do not differ and appear to be quite comparable. For example, the age of 80% of the general rural population ranged from 20 to 40 years old, a young population in their productive years, which was similar to our findings. On the other hand, in terms of sex, the composition of the general rural population was mostly male, unlike our results in which the majority was female. This can be explained by the fact that our survey studied the distribution of sex of the informants only and not the composition of sex within each household.

In terms of types of housing, our results differed from the ENDS survey, where ENDS reported that the majority of the general rural population lived in houses, while the majority of our population reported living in other types of structures. This difference is explained by the fact that the population of ex-combatants were located in the ETCRs, which have characteristics that are different than the rest of the rural population. Lastly, the two surveys also differed with regard to highest educational level, with the highest grade being incomplete elementary school for the general rural area and incomplete high school for our population. These differences may be due to our study having characterized the educational level of the main informant only and not that of the entire family. With regard to emergency services over the previous four weeks, the present study reported 17.4% usage of these services, and 75.2% of those who sought emergency services went to the local hospital. This is in agreement with the general rural area report by the 2015 ENDS. For both surveys, the populations in these regions were healthy young adults, which is also supported by our findings that obstetric care was the main reason for seeking emergency services. With regard to people with a health problem who did not seek health care services, the percentage was lower in our study (3.4%) than in the ENDS survey (42%), which had a high proportion for the general rural area reportedly due to the health services themselves (32.1%). While our survey did not directly ask why people did not seek care, it did show that a very small percentage did not seek care, and that the reasons were not related with the services offered in the area but rather with not being enrolled in the health system.

With regard to paying for the health services received, our findings are similar to the 2010 ENDS survey ^29^, which asked this question in relation to emergency services and found that a high percentage of the rural population reported not having incurred additional health care costs when using those services. This may be because most of the care provided was not complex and the costs were paid by the benefits plan, which is why Colombia has one of the lowest out-of-pocket health care costs in Latin America. Studies of other countries, such as Mexico’s 2016 ESANUT survey, have also reported that those enrolled in state or public insurance systems tend to pay little for emergency and out-patient services ^10^.

Nevertheless, the present study found that the EPS did not provide all the medications, which required people to make out-of-pocket purchases. The ENSANUT in Mexico had different results, where nearly 77% of those enrolled received their medications and did not have to pay out-of-pocket ^10^. Therefore, our country continues to experience difficulties with the availability of medications and access to them in rural areas.

With regard to the perception of emergency health services, it is worth noting that the national and international sources that were consulted did not report specific comparable results as to whether the care received was respectful or culturally appropriate, or whether the same service providers would be used when needing health services again. In terms of access, both of those factors relate to acceptability and satisfaction, and in both cases our study found that high percentages of our population of ex-combatants and their families had favorable perceptions of the emergency care that they received.

In conclusion, in spite of the limitations in availability and access to medications, the population of ex-combatants who received emergency services generally considered the quality of care given at the local hospitals to be good (60% of the participants in the study). This is similar to that found for emergency services in Mexico, which also reported a good rating in the majority of cases (60.34% of those interviewed) ^10^.

With regard to perception of access to prevention and promotion services and extramural activities for our study population, only 53% of the total number of households surveyed had some kind of contact with promotion and prevention services, which was because of spontaneous demand more than induced demand. Our study also found reports of little access to these activities by both specific high-risk groups, those under 1 year of age and pregnant women. It is also worth noting that other age groups also reported not having participated in these types of activities.

The findings were similar for extramural activities offered by the Health for Peace project, with a small percentage of families having received visits (17%), although a large percentage of households knew about the existence of these extramural teams.

Based on these findings, certain situations that are occurring with health services and the community in the territories can explain what has been found. One is related with the logistics and dynamics that are involved in providing health services, namely, induced demand as a strategy to promote not only activities required by law but also those that are voluntarily offered in order to strengthen primary health care (PHC), such as extramural activities. Induced demand is defined as all the actions that are aimed at informing and educating the enrolled population in order to deliver the activities, procedures, and interventions for specific protection and timely detection, as established by the technical guidelines. The Health Promoter Entities, Adapted Entities, and Administrators in the Subsidized System shall develop and implement strategies that ensure that their enrollees have access to procedural activities and to timely specific protection and detection interventions, as well as to health services for diseases that are of interest to public health, in accordance with age, sex, and health conditions ^27, 30^. The other situation is that health services are not being widely offered to the population of ex-combatants, thereby affecting both their availability and access to them. These two dimensions can be understood as follows: if they are not offered, then the services will not be known, and thus, they will not be demanded or utilized.

In terms of comparing our results on the use of promotion and prevention services in Colombia, no studies of urban areas with findings similar to ours were found. And although the results from urban regions correspond to socioeconomic and cultural contexts that are different than the present study, we are presenting two studies for the purpose of comparing possible similarities and differences in effective access. One of those studies was performed in Manizales, which was a household study of users in the contributory and subsidized systems that was aimed at determining the factors that influenced the use of promotion and prevention programs. In general, it found the frequency of use of those programs to be low, with only 38% of the households surveyed knowing about them. For those who knew about the programs that were available in the subsidized system, vaccination was most well-known and activities for adults were least well-known ^31^. Another study with a similar aim was performed in Medellin, which evaluated the barriers and strategies related to different actors having access to promotion and prevention services. It found that “the users’ lack of knowledge about their rights and responsibilities is a barrier to accessing health services, a lack of information results in users not requesting services about which they do not know they have a right, or in activities involving others that saturate the system,” such as overuse of emergency services ^32^. That supports our findings on promotion and prevention services and extramural activities for the population of ex-combatants, with similar percentages of offerings and access for those under 1 year of age and pregnant women, in which knowledge about these programs promotes soliciting them and actual contact coverage.

Although our survey found that access to these services was low, it is important to mention that the people who received health care indicated a high positive perception of availability, accessibility, contact coverage, quality, and satisfaction with both services (prevention and promotion, and extramural services). The evaluations by the ex-combatants mentioned that appointments were given within a maximum of 1 day, they received good treatment in accordance with their culture and beliefs and good care with relevant recommendations, and that they would use the services again. A study performed in Manizales described similar evaluations of the quality of service and relevance of promotion activities, where overall satisfaction was 56% in spite of low use and poor knowledge of the services ^33^, compared to 69% on the part of ex-combatants for promotion and prevention services and 70% for extramural activities. Thus, while these services are technically formulated to meet the health needs of the population, actions are needed in order to potentialize demand, outreach, and knowledge about these services, since that is the most effective way for the population of ex-combatants to exercise actual access to them.

Another less obvious situation indicated by our findings is that the population did not spontaneously access the services. This should be taken into account, especially since the survey’s data indicates that there was more knowledge about the presence of extramural teams in the territories than there was contact with them. Two reasons can explain this. One is a lack of information that would enable them to know that they could access these services, as explained previously. The other is a very weak or unrecognized capacity for self-agency in health. Similar findings have been reported by other studies of access to primary health services in rural areas, and are supported by the *health belief model*. Composed of six dimensions related to the person (perception of susceptibility, severity, benefits, barriers, self-efficacy, and cues to action), this model enables identifying the degree of importance or value that a person assigns to caring for their health. It reflects one’s own conception, independently of the health system ^33^. A study performed in Ethiopia used this model to evaluate the determinants involved in rural adolescents using sex and reproductive health services. It found that the characteristics or dimensions that most impacted interest in accessing those services were: the adolescents having at least a ninth grade education, discussing these topics among the family, having a high perception of the severity of the health problems related to this component, and a perception of great benefits and few barriers in terms of accessing these services ^34^. Two other studies determined barriers to health access, one in Uganda with adolescent mothers and another in Pakistan with rural pregnant women. The problem matrix in each of these studies showed that individual barriers were most important, including lack of knowledge about self-care practices and insufficient health education in school. With regard to social barriers, health services were not sought due to social stigmatization related to teenage pregnancy, including at school. Family barriers included the partner not permitting access to health services or family opposition to the health practices performed in hospitals and their preference for traditional health practices ^34, 35^. These studies show that even when services are available and offerings are sufficient, something inherent to the person will always enable or prevent someone from accessing health services or seeking advice, which cannot be controlled by the system, but rather, depends on the beliefs and perceptions of the individuals themselves.

With respect to the finding of a lack of knowledge about the project’s community health monitoring component that was developed for the population of ex-combatants, the reasons for this again pertained to low induced demand, or poor outreach, thereby reaffirming that deficient outreach by the programs or strategies being developed is the main barrier to accessing them. A similar finding was reported by the Medellin study mentioned earlier ^32^. This has a much greater impact in rural areas such as those in the present study, where a dispersed population makes health communication even more difficult. In fact, when asking the population of ex-combatants if community health monitoring strategies were important to accessing health services, the response was resoundingly positive, supporting what some social leaders have expressed in other studies conducted in the country, that “the best way to bring people into health services is to include them in an organized way so that they actively participate in improving the health system” ^32^. Along these same lines about the important role of community health monitoring in the perception of access to services, it is worth mentioning what is known in public health as community-oriented primary care, as presented by Gofin, which highlights the importance of this type of strategy because both health services and the population assume responsibility for the health of the community, which the community also identified as important. By establishing these networks, demographic and health monitoring can be ongoing and both parties can participate in evaluating local action programs, screening the population, and assessing interventions at the individual, family, and community levels. In this way, clinical health care for individuals and families can be integrated into public health ^36^.

Paraguay and Spain have demonstrated success with implementing these types of strategies in rural communities, one through surveillance and immediate health care for fever cases and the other through improvements in the quality of health promotion by facilitating the community’s involvement in the process, not only as reporters of the events of interest but also as community health agents. Through this strategy, the community has also been able to play a crucial role in identifying needs and formulating health promotion strategies, as well as detecting people with health risks and referring them to health services. This has improved morbimortality indicators for both case studies, in Asunción (Paraguay) and in Aragón (Spain) ^37, 38^, and supports the high interest that stood out in the results from the present study. Informed of these types of initiatives and involved in them, the community of ex-combatants would first consider the health benefits that would be gained from their implementation, and second, actively participate.

The perception of access to health services on the part of the population of ex-combatants who were surveyed may have been affected by various factors related with the logic by which the demand and supply of health services in the country are organized. One important and indicative factor in the dynamic of these communities may often go unnoticed —the *rural areas* themselves, where they are located. This is the case not only for ex-combatants in the process of reincorporation but also for a large part of the country. When health services planning does not take into account the rural context of the territories and the great importance of guaranteeing effective access, it could be said that the dispersion of the population ends up acting as an intermediary in creating barriers that make it difficult to provide services to the population. This produces problems in the perception of availability and accessibility, and results in a percentage of the rural population’s demand for health services not being effectively addressing, subsequently worsening their health conditions ^39^.

This type of geographic isolation can spiral into all types of marginalization —economic, political and social— which operate as indirect determinants of the lack of access to basic community services, or the exclusion from the right to enjoy them. Even though physical-natural adverse conditions of place are considered to be the causes of isolation, intrinsic conditions should not result in a failure to take public policy actions to alleviate a situation that is caused by segregated spaces, where social goods such as health are not easily accessible ^40^.

Other studies in Colombia have described similar differences in access to health services among regions, such as one by the Bank of the Republic in 2014 that compared the 2007 and 2012 quality of life surveys ^41^. The main difference found by that study was that the percentage of people with the least access to health care were located in Orinoquia and Amazonia, which are the most remote regions in the country. These disparities are primarily associated with the unequal distribution of hospital services, which are primarily located in the largest cities where the largest number of providers per 100,000 residents are found. Some of the main barriers to services were low perception of the risks caused by health problems, a lack of financial resources for traveling, and a perception of poor service. Paradoxically, enrollment increased during those years, particularly in the subsidized system, while the guarantee of access to services declined. Lastly, regression models to determine the probability of accessing services by region found that the probability of accessing services was lower in the Pacific coast and Orinoquia-Amazonia than in the other regions, primarily because few health providers are located in those regions ^41^. These regional disparities within the same country are of great concern when evaluating perception of access. Rural coverage under these conditions ends up in “no man’s land,” as Borgia said in his study of rural health care in Paraguay, who noted that the government’s recent health reform lacked definitive mechanisms for providing health to the rural population and made the role of rural community clinics invisible ^42^.

In our country, concerns over differentiation in access to services in these types of regions suggest some urgency to develop and establish a rural health model ^5^, planned in accordance with the perspective of each region so as to not only solve health problems in a timely way and with quality care but also to contribute to improving the living conditions of those located in these areas of the country (as described in point 1 of the peace accords). The Health Project for Peace is an important step in access to services for the population of ex-combatants.

This study has some limitations. First, it is a measurement of perception, we assume that the responses are loaded with subjectivity and may differ from objective measurements, for example when measuring waiting times. However, we are convinced that this measurement produces a result closer to the experience lived by the subjects when they need and seek medical attention. Second, we could not carry out a simple random sampling. However, and as indicated in the methods, the results are free of bias to the extent that 95.8% of the ETCRs of Colombia were surveyed (23 of 24), in some cases all the households of the ETCR were included, and mainly, due to the high homogeneity in the living conditions of the subjects.

The sampling design used by the present study does not permit making inferences at the ETCR or regional level, or comparisons among them. The regional stratification presented in the tables is only exploratory. It is to be expected that differences in the sociocultural characteristics of the population can affect the perception of effective access, as can differing degrees of success with implementation across the country and satisfaction with the services at the territorial level. Nonetheless, this is not something that can be evaluated or concluded based on the results of the present study. Valid information about the status of the implementation of the Health for Peace project in each ETCR or region did not exist at that time, nor does it exist now. In order to identify territorial differences, a different type of analysis needs to be performed using different methods and sources of information. While an overall analysis of the status of the implementation of the Project is undoubtedly research that needs to be conducted, it goes beyond the scope of this study.

Finally, there is the lack of generalization. The results of this study cannot be generalized to the total population of Colombia, nor to all those demobilized at the different moments of reincorporation processes during the internal armed conflict of the country, insofar as it is a population in special conditions, in transition to the legality and in the process of change in multiple dimensions; In this regard, we recognize that the perception of former FARC-EP combatants about the national health system is developed in comparison with the conditions of medical care during their militancy period before the signing of the Final Accord.

As a practical and experiential experience, it has played a positive role in the majority of the population having positive perceptions, as found by our study. These actions have indirectly strengthened the health dimension component of the path to reincorporation, which guarantees this fundamental right and recognizes this population as citizens who are transitioning to civilian life, thereby ensuring the conditions that are needed for a stable and lasting peace. It is important that these types of strategies and actions be sustainable and be extended to the entire rural population through a national plan and state policies aimed at making actual access to health services possible. In practical terms, this is the only mechanism that will subsequently transform the current perception of access to health services.

## Conclusions

The actions that have been implemented through the Health for Peace framework, which inspired this study, have taken into account WHO recommendations for health equity and universal health coverage for highly vulnerable populations ^43^, such as ex-combatants in the process of reincorporation. These two conditions are operationalized by effective access to services through strategies and actions that involve primary care in rural areas. In this way, it is possible to strengthen actual access to services for this population and go beyond the legally-required intra-institutional actions, towards extramural actions and community participation, which as documented herein, have a stronger impact on the highly positive perceptions of the majority of our study population. More of these actions are needed since they reduce the gap between rural areas and cities, and enable ex-combatants to put down roots in civilian life, thereby helping to maintain an extended post-conflict period that can lead to lasting and stable peace.

## Data availability

### Underlying data

The database is stored by the International Organization for Migration’s (IOM) Migration and Health program. Since it contains data about people who are ex-combatants and residents, this database cannot be publicly provided without prior authorization from the administrators, who will evaluate the request. A reviewer or reader interested in acquiring the data should write a request to the Migration and Health Program of the International Organization for Migration in Colombia, specifying: objectives, justification, expected results and dissemination plan of the analysis. Please send your requests to the director or the Program David Rodríguez **: darodriguez@iom.int**

### Extended data

Figshare: Instrument in Spanish of the Study “Perception of effective access to health services in Territorial Spaces for Training and Reincorporation, one year after the peace accords in Colombia: a cross-sectional study”, https://doi.org/10.6084/m9.figshare.11336468 ^14^

This project contains the following extended data:

- InstrumentinSpanishPerception of Effective Access to Health Services in Territorial Spaces for Training and Reincorporation.xlsx (Study instrument in Spanish)

Figshare: Instrument in English of the Study “Perception of effective access to health services in Territorial Spaces for Training and Reincorporation, one year after the peace accords in Colombia: a cross-sectional study”. https://doi.org/10.6084/m9.figshare.11328344 ^15^

This project contains the following extended data:

- InstrumentinEnglish.xlsx (study instrument in English)

Data are available under the terms of the Creative Commons Zero “No rights reserved” data waiver (CC0 1.0 Public domain dedication).
