# Supplementary material for: Perception of effective access to health services in Territorial Spaces for Training and Reincorporation, one year after the peace accords in Colombia: a cross-sectional study
Source: F1000Res. 2020 Jun 9;8:2140. Originally published 2019 Dec 23. [Version 2] doi: 10.12688/f1000research.21375.2 (PMC7331105; doi:10.12688/f1000research.21375.2)
Supplement: Peer review history

# Peer review: Reviewer response for version 2

**DOI:** 10.5256/f1000research.26646.r64491

**Published:** 2020-07-01

**Authors:** Cesar Abadía-Barrero, Vanesa Giraldo

**Affiliations:** 1 Department of Anthropology, University of Connecticut, Storrs, CT, USA; 2 University of Massachusetts, Amherst, MA, USA

We appreciate the authors’ responses to our questions and suggestions.

1. The conclusion in the abstract does not match the conclusion in the article.
2. The health belief model is inappropriate to explain why people do not spontaneously look for healthcare services. The results do not provide any data that allows to suggest a “weak or unrecognized capacity for self-agency in health” and do not evaluate any of the six dimensions of this model (perception of susceptibility, severity, benefits, barriers, self-efficacy, and cues to action) to understand the degree of importance that ex-combatants assign to their own health. In addition, the cited works about the health belief model are not related to the topic or the findings of this research. A more plausible explanation could be related to the transitional moment in which ex-combatants are starting to interact with a healthcare system different from the one they had as an armed group.
3. The new references about rural health provide interesting insights to understand the results
4. The article would benefit from a final proofreading

Is the work clearly and accurately presented and does it cite the current literature?

Partly

If applicable, is the statistical analysis and its interpretation appropriate?

Not applicable

Are all the source data underlying the results available to ensure full reproducibility?

Partly

Is the study design appropriate and is the work technically sound?

Yes

Are the conclusions drawn adequately supported by the results?

Partly

Are sufficient details of methods and analysis provided to allow replication by others?

Partly

Reviewer Expertise:

Medical Anthropology, Human Rights, Colombia

We confirm that we have read this submission and believe that we have an appropriate level of expertise to confirm that it is of an acceptable scientific standard.

# Peer review: Reviewer response for version 2

**DOI:** 10.5256/f1000research.26646.r65567

**Published:** 2020-06-26

**Authors:** Diego Lucumí

**Affiliations:** 1 School of Government, University of Los Andes, Bogota, Colombia

In my opinion, this is a well-written paper. Although the study is descriptive in nature, it provides relevant evidence about a recent public health topic in the Colombian literature. In this regard, I recommend the authors provide some potential areas of research that need to be addressed to extend evidence on this relevant area. For instance, based on the descriptive findings, some hypothesis could be proposed.

 In addition, I suggest to review some minor aspects:

- Check the order of the education category in table 1 (completed high school has to be the last category).
- It is clear there are few comparable studies on this topic. However, it could be relevant to see if, for example, for out of pocket payments it is possible to compare with reports from rural areas in Colombia.
- Undoubtedly, perceptions play a key role in explaining and influencing how people relate to health providers. I am wondering, why in this study authors recognize the role of theory and models as the Health Belief Model, but they decided to use a more general model to guide the conceptualization of the study. It does not invalidate the study but could be interesting to see if models that address specifically perception can lead to similar to different results.
- Finally, I suggest trying to reduce the extension of some paragraphs and the discussion section itself.

Is the work clearly and accurately presented and does it cite the current literature?

Yes

If applicable, is the statistical analysis and its interpretation appropriate?

Yes

Are all the source data underlying the results available to ensure full reproducibility?

Yes

Is the study design appropriate and is the work technically sound?

Yes

Are the conclusions drawn adequately supported by the results?

Yes

Are sufficient details of methods and analysis provided to allow replication by others?

Yes

Reviewer Expertise:

Social determinants of health and health equity, Cardiovascular disease prevention, Public health management.

I confirm that I have read this submission and believe that I have an appropriate level of expertise to confirm that it is of an acceptable scientific standard.

# Peer review: Reviewer response for version 1

**DOI:** 10.5256/f1000research.23542.r58511

**Published:** 2020-03-25

**Authors:** Cesar Abadía-Barrero, Vanesa Giraldo

**Affiliations:** 1 Department of Anthropology, University of Connecticut, Storrs, CT, USA; 2 University of Massachusetts, Amherst, MA, USA

This study addresses one of the most challenging dimensions of the implementation of the peace agreement in Colombia: access to health care services of former combatants. Using a human rights framework, the article aimed to describe how ex-combatants of the Revolutionary Armed Forces of Colombia-People’s Army (FARC-EP), now a political party, perceive access to emergency services, and health promotion and illness prevention programs during their reincorporation process to civil society. All participants inhabited one of the Territorial Spaces for Training and Reintegration (ETCR) which are places established to relocate former fighters. Developing instruments to monitor the socioeconomic dimensions of Disarmament, Demobilization, and Reintegration (DDR) operations, in particular, access to health care services, is not only an important contribution for Colombia, but for all countries that are transitioning from war and conflict to peace. This study also suggests an important contrast between access to healthcare in ETCRs and historical challenges for health provisions in rural areas. While the study is clearly of utmost importance, we identified several areas that warrant significant revisions. 

**Introduction:**

 The introduction provides limited context about the reincorporation program, ETCRs, and Health for Peace project. Although Colombia has had multiple experiences of reintegration programs with different armed groups, including the FARC, the process that followed the signing of the 2016 peace agreement is a unique experience that represented a national and international precedent for peace negotiations. The article should mention the main characteristics of the reincorporation program, including the different challenges and developments in the establishment, transformation, and infrastructural conditions of ETCRs in order to understand the living conditions of the participants. There is an important history here that should be mentioned, even in abbreviated form. Regional differences and successful and less successful ETCRs should be briefly mentioned as a way to convey the potential heterogeneity of experiences of ex-combatants. The authors refer to this program as reintegration; however, the distinction between reintegration and collective reincorporation is necessary in the Colombian context since it refers to different programs that determine ex-combatant lives in different ways. 

 Also, the authors do not provide a description of the Health for Peace project. Therefore, it is difficult to know how the intervention of United Nation agencies, the International Committee of the Red Cross, and other medical humanitarian agencies created special conditions in ETCRs. For the context of the present study, it would be important to clarify what were the actions implemented by this project? Did they offer healthcare services or programs? Did they help ex-combatants with the administrative processes of the Colombian healthcare system? Was there a transition or potential overlap between international agencies and the Colombian health care system? Were there services from the Health for Peace project, including ambulances, physicians and nurses, still present at the time of the study? If there was overlap and the agencies were still present in the ETCRs, is the study instrument able to differentiate between the actions implemented by these agencies and the professionals and networks of the Colombian healthcare system? A more extensive description of this historical context of the relationship between the ETCRs, the international humanitarianism that supported the reincorporation process, and the health care networks is very relevant for the comparison with other rural communities and other peace processes. 

 For an international audience, the concepts of “prevention and promotion” and “extramural activities” should be briefly explained.

**Methods:**

- The methods are appropriate to answer the question of the study.
- The high coverage of the sample and the number of ETCRs included are remarkable given the characteristics of these population. 
- The study unit seems to be the household not the family.
- It seems that the authors overlooked the name of the survey developed by Arrivillaga, Aristizabal, and Estrada and wrote instead East Asian Social Survey.
- The instrument has an important merit for this kind of study; however, one of its limitations is that it does not include questions about conflict-related health issues, focuses primarily on emergency services and only assesses the four weeks prior to the survey. This should be taken into account in the analysis and discussion.   

**Results:**

 The core of the results of the study is presented in tables and figures. There are problems in the way the data are reported and we question whether the authors need to present all these data and whether some data deserve further analysis. 

**For both tables:**

- It is customary to present results as n (%). Since totals are numbers, it would be preferable to have the tables presented as n (%).

**Table 1:**

- State that CV is coefficient of variation.
- Place of Residence. It seems that this category was transferred from other studies. However, it is not clear how the category ‘urban’ applies to the context of ETCRs. Furthermore, the differences between rural settlements and dispersed rural areas are not intuitive and deserve an explanation.
- Number of people in household. There seems to be a problem in the reporting of the total column. The total for mean and median doesn’t match the addition of men and women.
- Cultural identity or physical trait. Please change heading to Cultural identity and/or race/ethnicity.
- We recommend a table that shows the distribution of identity/race/ethnicity among the regions so we can have a better sense of the relationship between these two variables (Region and cultural identity/race/ethnicity).
- Group. The definition of the categories should be explained. For instance, what is the difference between victims and displaced? The authors should also mention that respondents could mark several responses.
- Housing. ETCRs, as part of the settlement project of the peace accord, have very similar characteristics. These categories, however, do not seem to reflect the housing conditions of ex-combatants in the collective reincorporation program. We suggest explaining the general characteristics of these types of housing. It seems that some questions were also adapted from other survey studies and it is unclear how they were transferred to the context of the ETCRs. For example, it is hard to think how “rented room” (inquilinato in the original instrument) is even a possibility within the context of ETCR. Equally, it is unclear what “room in another type of structure” refers to. 
- Role in the family. In this question, if all the interviewees were the heads of the household as it is reported on page 3, it is unclear what relationship categories represent.

**Table 2:**

 This table presents the most interesting results of the study. Nonetheless, the variations between regions deserve further analysis and discussion. Importantly, the Pacific and the Amazonian region show important differences from other regions. Depending on the final results and final presentation of the table, relevant discussions about regional variability could take place. In particular, the reader wonders why these two regions rank poorer in people’s perception of effective access. There can be interesting discussions with other Colombian studies around health inequalities and regional variation and this study opens up the possibility to discuss what is specific of the peace accord and what resembles larger problems of the population of these areas. 

 Given the time the data were collected (September and October 2018), we know that some ambulances and health care personnel were still present in some of the ETCRs. Then, it is not entirely clear if the services people received were part of the Health for Peace project that had significant funds from international cooperation or if people were, in fact, using the services of the healthcare system. If ambulances were available, did that facilitate access to services and in a way skewed the data? Further information of the historical moment and specific conditions of the ETCRs at the time of the interview, as mentioned for the introduction, could help with the analysis of these results. 

 Figure 1, 2, and 3 offer interesting information but it is very hard to assess the responses. Are there national and international guidelines that allow us to assess whether those percentages are adequate? Furthermore, Figures 2 and 3 require information about the clinical specificities of the children under 1 or pregnant women to assess whether the services offered were adequate or not, perhaps according to whether their regular or schedule check-ups are in agreement with known guidelines. We are left wondering how important this information is. The fact that the questions ask for the last four weeks further complicates the analysis given that the person could be in a moment in which there is no need to use these services and appointments might have already happened before the four weeks period or are scheduled for after the interview.  

 Figures 4 and 5 would benefit from an explanation of these programs and the guidelines or parameters established in the country regarding their offering. Are health promotion and illness prevention services replacing regular care? Why would someone have to schedule such an appointment rather than already having an appointment or being contacted to schedule the appointment? How do referrals and enrollments in these programs work? Also, questions arise about the coordination between programs and whether the presented data is adequate or follows any established guidelines. 

 All in all, an explanation of the ways in which prevention and promotion, extramural services, surveillance and monitoring systems, and regular health care work in coordination is needed to make sense of figures 1 through 8. Then, the authors should conduct the analyses of these data based on that description of the multiple pieces of the health care system and the parameters used to assess how adequate or inadequate are. Just to mention one example, what does it mean that 17,3% of the sample said that they had contact with an extramural team over the past 4 weeks? Is this adequate, inadequate, desirable, worrisome? We encourage the authors to rethink the presentation of the results.  

**Discussion:**

 The discussion requires major changes. The authors discuss some of the results in relation to existing literature but it is unclear what are the central ideas of the discussion and what results the authors found particularly interesting and relevant.

- Page 13. 2nd paragraph. “While prioritizing the right to health for ex-combatants represents important progress in public policy, Colombia’s health system faces numerous challenges that need to be studied”. What challenges is this sentence referring to?
- Page 13. 3rd paragraph. The ARN survey of 2014 describes a different reintegration program. This program includes ex-combatants who demobilized before the peace agreement and the majority of them moved to urban areas. As mentioned, the article should specify from the beginning that the population assessed in the study are part of the collective reincorporation program and describe their main characteristics. Is it possible to discuss some differences between access to health care depending on whether the ex-combatants participated in the reintegration or the reincorporation programs?
- Page 13. 1st paragraph. “ (...) the results from the present work provide a new methodology and information that can be used to identify the health status of populations that became invisible because of their situation as active actors in armed conflict.” This phrase is unclear. The present study centers on ex-combatants once they became “visible” and legally recognized after the peace accord. It also has the limitations of asking about the four weeks prior to the interview. 
- The contrast with healthcare provision in other rural areas is an interesting and important discussion. However, the main points of this contrast are not clear. 
- Page 14 3rd paragraph. We suggest avoiding adjectives such as “high”, “low” and “small” without providing specific data.
- The discussion of the results about health promotion and illness prevention services in relation to studies conducted in Manizales and Medellin does not seem appropriate, unless the difference between these services in rural and urban areas is stated.  
- What do the authors mean by good treatment in accordance with their culture and beliefs? Are the notions of culture and beliefs related to their identity as ex-combatant, a regional identity, a rural identity, or an ethnic/racial identity? 
- The results do not indicate that people’s behavior to seek health care services can be explained through the health belief model.
- We suggest that the center of the discussion should be around how the current data compare to the challenges described for other rural populations. The authors hint in this direction but could make significant contributions by signaling how ex-combatants’ perception of their access to health care compares to other studies in rural areas. Are they getting better or worse access to health care than other rural communities? What does this say about the reincorporation process and about the health care system? If the analysis is broken down by region, what does it tell us about different regions in Colombia and, perhaps, different “reincorporation processes”? 

Is the work clearly and accurately presented and does it cite the current literature?

Partly

If applicable, is the statistical analysis and its interpretation appropriate?

Not applicable

Are all the source data underlying the results available to ensure full reproducibility?

Partly

Is the study design appropriate and is the work technically sound?

Yes

Are the conclusions drawn adequately supported by the results?

Partly

Are sufficient details of methods and analysis provided to allow replication by others?

Partly

Reviewer Expertise:

Medical Anthropology, Human Rights, Colombia

We confirm that we have read this submission and believe that we have an appropriate level of expertise to confirm that it is of an acceptable scientific standard, however we have significant reservations, as outlined above.

# Response

**Published:** 2020-05-13

**Authors:** Julián Alfredo Fernández-Niño

**Affiliations:** Universidad del Norte, Colombia

**Answers Observations, Questions and Requests**

 

1. **Introduction:**The introduction provides limited context about the reincorporation program, ETCRs, and Health for Peace project. Although Colombia has had multiple experiences of reintegration programs with different armed groups, including the FARC, the process that followed the signing of the 2016 peace agreement is a unique experience that represented a national and international precedent for peace negotiations. The article should mention the main characteristics of the reincorporation program, including the different challenges and developments in the establishment, transformation, and infrastructural conditions of ETCRs in order to understand the living conditions of the participants. There is an important history here that should be mentioned, even in abbreviated form.

**RTA:**

In the discussion, we had described the general characteristics of the elements of the reincorporation program that are related with access to the health system, and the relevant components, including a summary of the components of the process, along with some of the challenges for organizing social services, and particularly the right to health and the provision of health services for ex-combatants given the conditions of rurality itself in Colombia. This was aimed at providing the reader with greater context for interpreting the findings. To address the reviewer’s observations, we adapted two paragraphs in the discussion and moved them to the introduction in order to provide the reader with this context at the beginning of the manuscript (new paragraphs 4 and 5 in the introduction):

“The Colombian government established eight dimensions for the path of reincorporation, one of which is access to the General Social Security Health System (SGSSS, in Spanish). It also established health services as a fundamental right to be guaranteed along with returning these people and their families to legality. Colombia is a good example of the comprehensiveness that these programs need when it comes to ex-combatants who are on the path to reincorporation, in the short-term. Meanwhile, long-term and sustainable reincorporation into civilian life will be ensured by including crucial elements such as effective health care and ongoing access to health services through enrollment in the health system. This will contribute to individual well-being, continued legality, and a solid ground on which to build stable and lasting peace.

Some of the projects were developed with the above challenges in mind and in accordance with the reference framework on which the path was laid out, such as Health for Peace, led by the Ministry of Health and implemented by three United Nations agencies (UNFPA, PAHO/WHO, and IOM). This project was created in order to strengthen institutional and community capacities to address health needs in the rural areas where the ETCRs are located, through already-established regional health centers as well as local primary hospitals. Health services were also complemented by the development of extramural strategies and community surveillance, led by regional health professionals with the support of local public health leaders who were trained by SENA as part of the project”.

SENA is the National Learning Service (Servicio Nacional de Aprendizaje), which is the main public institution in Colombia responsible for technical training at the national level.

In addition, to addressing the reviewers’ suggestions, a new paragraph was included in the introduction (new paragraph 6 in the introduction). We believe this provides the relevant context from which the survey used in this study arose, more specifically, in the framework for the implementation of the final accord. We briefly mention the Health for Peace project, its coordination with the health system, and the historical change that this represented for the organization of health services in the ETCR:

“In this way, the “Health for Peace” contributes to the implementation of Point 1 in the Implementation Framework Plan (IFP) of the Final Accord, which relates to comprehensive rural reform and is associated with the implementation of Territorial Health Plans in the priority municipalities. It also complemented the emergency response by the Colombian government during the first year after the accord, which consisted of a physician, a nursing professional, and an ambulance for each ETCR. These health workers held positions that at first lacked the conditions needed to guarantee the quality of health care, given that the infrastructure and human skills for providing health services in the regions where the ETCRs are located has historically been insufficient, and non-existent in some cases. This emergency measure was gradually phased out as the Health for Peace project was being implemented. In this sense, the main objective of the Project was to strengthen the local capacity for improving access to comprehensive Primary Health Care services —with an emphasis on sexual and reproductive rights, mental health, preventing consumption of psychoactive substances, and children’s health and malnutrition— thereby addressing the needs that had previously been identified by the United Nations Verification Mission in the ETCR. Furthermore, in order to promote access to comprehensive health services, support was provided to implement an extramural strategy as part of Primary Health Care, as well as to develop a strategy for community participation and surveillance. This proposal can be considered to be the specific development of strengthening health services in the rural area through recent technical developments by the Ministry of Health, such as the National Public Health Plan, but adapted to the context of ex-combatants, their families, and the residents of these regions, with the advantage of receiving technical and financial support from agencies in the United Nations system.”

With regard to the other points mentioned by the evaluators, we believe that a more detailed description of reincorporation, the “history,” including ongoing challenges, cannot be summarized in only a few paragraphs without risking falling into debatable inaccuracies. This also clearly goes beyond the scope of the aim of our study, which involves a specific objective and moment in time, namely, to describe how ex-combatants perceived effective access one year after the signing of the accord. It is important to consider that, for a merely descriptive quantitative study, the article is already exceedingly long, and the analysis suggested falls outside the authors’ expertise. In addition, an extensive and more detailed discussion of the context is already included in the current version of the article, as well as potential contrasts with other reincorporation processes for ex-combatants in other countries.

Given our approach and disciplinary style, the objective of the introduction is to present the specific context that relates with the study. We believe that the most pertinent of what was suggested would be to add more information about the Health for Peace project, how that was implemented in the ETCRs, and where this project was inserted into the reincorporation process. We have added all of this to the introduction (paragraphs 3-5). More detailed information and analyses can be found in the references that are cited, especially in the discussion.

 

1. Regional differences and successful and less successful ETCRs should be briefly mentioned as a way to convey the potential heterogeneity of experiences of ex-combatants

**RTA:**

Agreed. Nevertheless, we believe the sample sizes in the ETCRs do not enable us to make inferences or draw conclusions about differences among regions or among ETCRs. The sampling was proportionally stratified by the size of the population of each ETCR for the purpose of making an overall inference only.

We included the regionally stratified analysis in the tables for exploratory purposes, as well as for reflection on the part of the readers and potentially for the formulation of a hypothesis. Nevertheless, we prefer not to speculate about whether the differences found are explained by differences in the program’s success, since they could also be due to randomness, which is why the discussion and conclusion about the results are presented using totals. We would certainly expect that there could be differences in the sociocultural characteristics of the population that could affect the perception of effective access, and that there could be differing degrees of success in implementation across the country. Nevertheless, this is not something that can be evaluated or concluded based on our results. We also do not have valid information from any other source about the status of the implementation of the Health for Peace project in each ETCR or region at that point in time.

In order to address the suggestion by the evaluators, we have added a paragraph that describes this aspect, but we prefer that it be presented in the discussion as a limitation, as follows:

“The sampling design used by the present study does not permit making inferences at the ETCR or regional level, or comparisons among them. The regional stratification presented in the tables is only exploratory. It is to be expected that differences in the sociocultural characteristics of the population can affect the perception of effective access, as can differing degrees of success with implementation across the country and satisfaction with the services at the territorial level. Nonetheless, this is not something that can be evaluated or concluded based on the results of the present study. Valid information about the status of the implementation of the Health for Peace project in each ETCR or region did not exist at that time, nor does it exist now. In order to identify territorial differences, a different type of analysis needs to be performed using different methods and sources of information. While an overall analysis of the status of the implementation of the Project is undoubtedly research that needs to be conducted, it goes beyond the scope of this study.

 

1. The authors refer to this program as reintegration; however, the distinction between reintegration and collective reincorporation is necessary in the Colombian context since it refers to different programs that determine ex-combatant lives in different ways. Also, the authors do not provide a description of the Health for Peace project.

**RTA:**

These concepts are used in accordance with the terminology in the documents that are cited. The International Organization for Migration (IOM) and the Ministry of Health, as co-authors, have asked us to use the same terminology in order to avoid ambiguity. We understand the debate underlying the distinction mentioned by the evaluators, and we suggest that the reviewers could present this in a public comment about the article, where it could be debated.

The Peace Accord is described in the brief introduction to paragraph 3, and a link has been added to the text where it can be consulted in full. The paragraph mentioned above (new paragraph 6 in the introduction) was also added, which describes the Health for Peace project and briefly explains the main components.

 

1. Therefore, it is difficult to know how the intervention of United Nation agencies, the International Committee of the Red Cross, and other medical humanitarian agencies created special conditions in ETCRs. For the context of the present study, it would be important to clarify what were the actions implemented by this project? Did they offer healthcare services or programs? Did they help ex-combatants with the administrative processes of the Colombian healthcare system? Was there a transition or potential overlap between international agencies and the Colombian health care system? Were there services from the Health for Peace project, including ambulances, physicians and nurses, still present at the time of the study? If there was overlap and the agencies were still present in the ETCRsA more extensive description of this historical context of the relationship between the ETCRs, the international humanitarianism that supported the reincorporation process, and the health care networks is very relevant for the comparison with other rural communities and other peace processes. 

 

RTA:

An “extensive” description of the historical context of the relationships between the ETCRs and international humanitarian organizations is relevant to this area of study, nonetheless, we believe that it considerably exceeds the scope and objectives of our study. This is not an objective of this report of descriptive information, nor is it of interest to the current research project. A different type of investigation would be warranted in order to respond to this aspect. The current manuscript is over 10,000 words, and we believe that elements that do not enter into dialogue with our specific results would not be relevant. Given our objective, we are unable to identify how it would enter into dialogue with the manuscript in general, or specifically with the results. While the context is undoubtedly relevant, this report has a modest objective, and its intention is not aimed at a historical analyses of the peace process. If the reviewers consider it to be helpful to suggest a reference that discusses this aspect, we can include that.

As we mentioned previously, our objective is limited to describing the perception of effective access to health services on the part of ex-combatants in the ETCRs, in the framework of the Health for Peace project. The elements that we consider to be relevant pertain to this context, and are presented in the manuscript, in the introduction and the discussion, and have been expanded. Adding broader elements would warrant a more detailed analysis using other methodologies and sources (sociological, historical, analysis of public policies) that go beyond the objectives of this report, and fall outside our disciplinary background. We believe that this is a task for experts on those types of analyses, who can use our results, among others, as a basis for their studies.

 

1. For an international audience, the concepts of “prevention and promotion” and “extramural activities” should be briefly explained

RTA:

We agree. And we would like to add that this article may also be read by people who have a background other than Public Health. Therefore, we want to take this opportunity to suggest incorporating a Glossary of Terms. We suggest including this as a separate linked file, or supplementary material, or as a table in the main article. We include it here in our response and we will defer to the editors with regard to where to place it.

This table is referred to in the methodology, specifically in the section “Data collection instruments and procedures”

**Glossary of terms**

**Health promotion**:  This is a global political and social process that encompasses not only actions aimed directly at strengthening the abilities and capacities of individuals, but also at modifying social, environmental, and economic conditions in order to mitigate their impact on public and individual health.

**Main Strategies:** To establish a healthy public policy, create environments that support health, strengthen community action for health, develop personal skills, and redirect health services (WHO,1986)

**Primary health care:** This refers to essential health care that is affordable for the country and the community, and it is conducted using practical, scientifically-founded, and socially-acceptable methods.

The primary health care approach includes the following components: equity, community involvement/participation, intersectorality, adequate technology, and affordability.

**Main activities:** Education on individual and community health, including the magnitude and nature of health problems, and indications for how to prevent and control these problems; supply of drinking water and basic sanitation; maternal and child health care, including family planning; immunization, proper treatment of common diseases and injuries; and the supply of essential drugs. (WHO, 1978)

**Disease prevention: **Measures aimed not only at preventing the onset of disease, such as reducing risk factors, but also at stopping its progression and mitigating its consequences once it occurs.

**Primary prevention** is aimed at preventing the initial appearance of a disease or illness.

**Secondary prevention and tertiary prevention **are aimed at stopping or delaying a disease that is already present, as well as its effects, through early detection and appropriate treatment or by reducing recurrence and chronicity, for example through effective rehabilitation. (WHO, 1984)

**Community action for health:** Refers to collective efforts by communities to increase their control over the determinants of health, and consequently, to improve health. (Otawa Letter, 1984)

**Extramural Activities: **This refers to services that are offered to the population in spaces not intended for health, or health spaces in regions that are difficult to access and where health services are not available. These spaces are temporarily setup for the development of specific activities and procedures. (Resolution 1441/2013 Ministry of Health Colombia)

**Methods:**

1. The methods are appropriate to answer the question of the study.

The high coverage of the sample and the number of ETCRs included are remarkable given the characteristics of these population. 

The study unit seems to be the household not the family.

It seems that the authors overlooked the name of the survey developed by Arrivillaga, Aristizabal, and Estrada and wrote instead East Asian Social Survey.

RTA:

Thank you very much for the valuable comments by the reviewers. Indeed the advantage of the study is its ability to encompass, with high representativeness, a population that is difficult to identify with this type of analysis. This was possible because of the availability of a sample of nearly all of the ETCRs that were active in the country at the time the study was conducted, and its availability at a critical time in Colombia’s peace process.

The unit of analysis is indeed the household, and not the family, which is why the description of the results refers to households rather than families.

In the second paragraph in “Data collection instruments and procedures,” we changed the name of the survey that was used. Thank you very much for the correction.

1. The instrument has an important merit for this kind of study; however, one of its limitations is that it does not include questions about conflict-related health issues, focuses primarily on emergency services and only assesses the four weeks prior to the survey. This should be taken into account in the analysis and **discussion**.   

**RTA:**

The main objective of the instrument that we constructed was to determine the perception of effective access to health services in a population of ex-combatants who were located in the ETCRs, and who had been given additional support through the provision of primary health and emergency services. The survey was not intended to establish an individual health diagnosis of ex-combatants in relation to their previous activity as combatants, but rather, to determine whether one of the strategies to guarantee the reincorporation of this population to civil society was met —that is, access to health services from the perspective of a right to health framework, which is a commitment made by Colombia’s government.  

Since we wanted to identify effective perception and not just access to services, an instrument was constructed to allow us to measure these dimensions in the population of ex-combatants who immediately received services. This was aimed at ensuring that the health services would have been provided primarily through the Health for Peace project. These specific actions were carried out within the framework set forth by Colombia’s health legislation, in order to help ex-combatants and their families access services and have their needs addressed, as well as to promote health. Therefore, by using a window of 4 weeks prior to the interview, we ensured that their answers corresponded to the care they were receiving at that point in time.

The study was not aimed at evaluating topics related with the conflict or access to health services by ex-combatants in the past or before the reincorporation process. The health services that were evaluated primarily correspond to the lines of action taken by the Health for Peace project, but also to public health actions taken in rural areas across the entire country. These were not limited to emergencies (which in rural areas tend to be the main point of access to health services), but also involved extramural activities, including Public Health actions and even community surveillance.

One month prior to the survey was chosen as the period since the information is cross-sectional and we wanted to use a reduced temporal framework that would be the same for all the study participants, such that we referred to a “snapshot” of what occurred for everyone during virtually the same period. A period of one month is usually standard for studies about health services. If we were to ask about health services over a longer period prior to the interview, the person would be reporting their perception of access at times other than the implementation of the project. A longer window could also result in recall bias.

This time frame is always indicated in the description of the results and the figures. And a brief explanation about the reason for using one month was added to the methods.

**Results:**

1. The core of the results of the study is presented in tables and figures. There are problems in the way the data are reported and we question whether the authors need to present all these data and whether some data deserve further analysis. 

**RTA:**

We believe that one of the main strengths of our study is the fact that we present an analysis of all the dimensions of the health services available at that time in the ETCRs, and that not including all of them would be exclusive. The research group’s efforts were directed at measuring all the dimensions of effective access, and the manuscript is aimed at a wide reporting of the results, which is common for a report of a multi-dimensional survey. This way, the readers can draw general conclusions and also extract detailed information on the dimensions that are most relevant to them. This can serve as a key database for more specific analyses by researches and decision-makers.

In our opinion, the results are easily readable. Other analyses can of course be performed with this information, for example, exploring associations among dimensions, but our purpose for this report is to provide a simple but exhaustive description that serves as a basis for researchers and decision-makers. There will be few opportunities to publish the complete findings, which is why we chose F1000research, so that the number of words would not be limited and to make this public quickly and openly.

Rapid and complete dissemination of the results would take full advantage of the resources used to collect the information, where knowledge management is very relevant.

Having said that, if the editor believes it is necessary, we could leave in the two tables, but perhaps dispense with some of the figures, although we think that the way they are structured would be helpful to decision-makers, who may want to extract the information for public presentations that would disseminate the findings.

 

1. **For both tables:**

It is customary to present results as n (%). Since totals are numbers, it would be preferable to have the tables presented as n (%).

**RTA:**

There are a variety of styles. We believe that % should be separate since it is the estimator of interest, as opposed to absolute frequencies, and it should be the first thing that the reader sees. We can change that if the editor believes it is necessary, but we prefer to keep it as is in order to stress that the results are interpreted based on these indicators (percentages), and especially when related to the conclusions.

1. **Table 1:**

State that CV is coefficient of variation.

**RTA:**

That’s right. We replaced CV with coefficient of variation.

1. Place of Residence. It seems that this category was transferred from other studies. However, it is not clear how the category ‘urban’ applies to the context of ETCRs. Furthermore, the differences between rural settlements and dispersed rural areas are not intuitive and deserve an explanation.

**RTA**:

All of the questions and categories included in the responses to the instrument, and therefore in the report of findings, were specifically designed and adapted for this study. In addition, the contents of the response options were validated in two ways: by experts and by the community. The latter included a review by field nurses who lived in the territory, as well as a pilot in which all the questions were reviewed at the territorial level, in order to study the acceptance, efficiency, and cultural applicability of each response. After completing the review, the option of “urban” was left in because the review found that it was applicable in some cases.

We used the definition of “urban” by the National Administrative Department of Statistics (DANE in Spanish), namely:  “It is defined as a concentration of a minimum of twenty (20) contiguous, neighboring, or semi-detached dwellings, located in a rural area of a municipality or a Departmental Township.”

In accordance with technical criteria, this aspect was studied and verified with the members of the team. Although there are few, the classification is applicable.

 

1. Number of people in household. There seems to be a problem in the reporting of the total column. The total for mean and median doesn’t match the addition of men and women.

RTA: No, the calculation that we present is correct. For the average of persons per household in the total sample, both averages (men and women) cannot be added (summed). It is important to take into account that we are stratifying by the sex of the informant (or head of household). Therefore, each column analyzes different households. That is, they cannot be added together, since what is presented is the average (and median) of persons in the household, for households with women as the head versus those with men as the head of household, as explained in the article. It’s helpful to remember that the unit of analysis is the household, as explained in the article, except that, here, head of household (or informant) data are presented as well as household data.

The mean of the total would actually be the result of an average weighted by the sample size of each group, as can be mathematically verified.

Cultural identity or physical trait. Please change heading to Cultural identity and/or race/ethnicity.

**RTA**: We changed it to “ethnicity”.

1. We recommend a table that shows the distribution of identity/race/ethnicity among the regions so we can have a better sense of the relationship between these two variables (Region and cultural identity/race/ethnicity)

**RTA: **We created the table that was requested, and we are including it below for the reviewer’s information. As can be seen, the ethnic distribution is as expected for the regions, although the ex-combatants are not necessarily in an ETCR that is in the same region as where they are from, which is an aspect that cannot be determined with the data that we have available.

**Distribution of ethnicity of the participants by region**

See the table in this link: https://www.dropbox.com/s/yozcvr3sidutvgg/Distribution%20of%20ethnicity%20of%20the%20participants%20by%20region.docx?dl=0

*Presented as absolute frequencies and percentages per column

Nevertheless, we do not believe it would be pertinent or congruent to include this table in the article, since it does not correspond with the objectives of our research. As we explained earlier, the sample design does **not** permit making inferences or comparisons at the regional level, but rather only global. The results in Table 2 are presented by region in an exploratory manner, but are household level data, not individual level. Thus, since the inference in the conclusions is at the household level, and we do not know the ethnicity of the entire family, then we cannot indicate a relationship between ethnicity and the variables of interest in the study that relate with perception of effective access by the household.

To summarize, the global description includes the ethnicity of all the informants in the entire sample in an informative manner, but it is not our interest to discuss explanations for regional differences. As we  mentioned, the sample design does not permit that analysis, and it is also not one of our objectives. In addition, the sample size does not enable us to explore differences among regions, and ethnicity  also cannot be connected with the results since the level of analysis is different (ethnicity at the individual level and effective access at the household level).

The table could be consulted in the public reviews, but it seems to us that its inclusion in the article is not warranted.

1. Group. The definition of the categories should be explained. For instance, what is the difference between victims and displaced? The authors should also mention that respondents could mark several responses.

**RTA:**

The authors did not use a working definition (theoretical or legal) of the categories in the present article. This work was based on a self-identification and self-affiliation approach, in which each person indicated whether they considered themselves a “victim” or “displaced.” This approach is relevant to these types of quantitative questions, and is even used for complex categories such as ethnicity.

Using a working definition defined by the authors would not be useful since the study participants themselves decided how they are classified based on their own criteria, which does not always correspond to theoretical and legal definitions. Thus, for example, “victim” or “displaced” is how one defines oneself. In addition, the methodology does not allow for determining labels in a distinct manner or with “objective” criteria, given that they are based on each individual case and only one simple question. (How it is formulated can be consulted in the questionnaire in Spanish, and its translation into English.)

Unfortunately, with regard to the question of group affiliation, at the request of the program, this was defined operationally and in an exclusive manner, though in reality the groups are not mutually exclusive (the same person could be in two or more categories) since people had to choose only one category with which they identified most. These groups were defined because they were the ones considered to be of interest to the program, and we found that the majority of the participants (over 71%) identified as ex-combatants.

All of this clearly indicates a limitation with respect to this variable. Therefore, these categories are presented as part of the characterization of the study population, but are not considered to be categories for analysis, nor are they intended to be more deeply explored. We included a comment about the definition of the groups in a footnote in Table 1.

 

1. Housing. ETCRs, as part of the settlement project of the peace accord, have very similar characteristics. These categories, however, do not seem to reflect the housing conditions of ex-combatants in the collective reincorporation program. We suggest explaining the general characteristics of these types of housing. It seems that some questions were also adapted from other survey studies and it is unclear how they were transferred to the context of the ETCRs. For example, it is hard to think how “rented room” (inquilinato in the original instrument) is even a possibility within the context of ETCR. Equally, it is unclear what “room in another type of structure” refers to

**RTA**:

This imprecision clearly stems from our original efforts to create transferability with some of the surveys that have been performed in rural areas in Colombia. We agree that it is not possible to consider whether something like “rented room” (inquilinato) exists in this context. The interviewers were consulted again about this question, and it was decided that all the cases were a type of rented room, while not being able to distinguish which type.

Those who indicated “rented room” were probably referring to a room in houses that were not their own and where other families lived, and that is what they called it (very few responded in this way). “Rented room in another structure” refers to a rented room in a house belonging to someone else, but that is not considered to be a rooming house. Therefore, we decided to unify “rented room” into one single category given that it is not possible to distinguish between these. For transparency purposes, we kept the original question as it appears in the instrument.

We do not have more information about housing conditions.

 

1. Role in the family. In this question, if all the interviewees were the heads of the household as it is reported on page 3, it is unclear what relationship categories represent.

**RTA:**

The concern about the role of the family was clarified in the methods section (paragraph 1), where the main informant was the head of household or the person who had the most information about the health status of the household members. Therefore, in the results table, we can see that the main informant’s role in the household was not only head of the family but could also be wife.

1. Given the time the data were collected (September and October 2018), we know that some ambulances and health care personnel were still present in some of the ETCRs. Then, it is not entirely clear if the services people received were part of the Health for Peace project that had significant funds from international cooperation or if people were, in fact, using the services of the healthcare system. If ambulances were available, did that facilitate access to services and in a way skewed the data? Further information of the historical moment and specific conditions of the ETCRs at the time of the interview, as mentioned for the introduction, could help with the analysis of these results

**RTA:**

We do not have this information and we would not be able to collect it in a valid manner.  We do not have records available to us that would enable us to answer this question. Since we only have oral reports from key actors rather than systematic and reliable data, we prefer not to include this. As we mentioned, the objective of this study does not include distinguishing between whether the given services were provided through international cooperation or by the healthcare system. In fact, for us, this difference is not very clear because international cooperation was aimed at strengthening the institutional response in a sustainable manner. Of course this is debatable, but we do not have more information with which to discuss it.

Below is an additional response that from the administrator of the IOM’s Migration and Health program, which could be published as part of the response, for readers who would like more discussion:

 “Yes, there were  ambulances in some of the ETCRs and health personnel who strengthened the hospitals of influence in the ETCRs. That was an intervention led and organized by national governmental agencies, as well as hundreds of interventions that were carried out to demobilize the ex-combatants. Comprehensive management of health care also existed by national agencies that played a role in complying with the peace accords. And there were calls for the presidency of the public insurance company to enroll ex-combatants, as well as many other interventions that affect perception. We do not believe there is bias precisely because what was measured was the perception of the multiple and specific social interventions in health that existed at the time. We are not saying that access to health by the rural population in Colombia was measured. We only have a snapshot of a single moment in the history of health in Colombia. It was not of interest to us to evaluate in a distinguishing manner which actions and institutions contributed to this perception.” We do not believe that it is relevant to include more details about coordination between international cooperating agencies and the Health for Peace project.

1. Figure 1, 2, and 3 offer interesting information but it is very hard to assess the responses. Are there national and international guidelines that allow us to assess whether those percentages are adequate?

RTA:

No standards for these indicators exist, and they are not easily comparable since they depend on demographic and epidemiological profiles, objective morbidity, perceived morbidity, self-perception of health, and the profile of the utilization of services, which are different for different populations and social contexts. The indicators in these figures are primarily a reflection of the offer and use of services, rather than the need, although that does partly determine usage.

Nevertheless, their publication does provide a reference for future studies and for making comparisons with similar populations.

This comparison is presented in the discussion, as is standard for similar publications. The discussion and analysis portion of the article compares our results with national and international parameters for perception of access to the different dimensions that make up this construct in the survey of emergency services, promotion, prevention, and extramural activities. The discussion describes the literature search that was performed and discusses the results in a detailed manner. The 2015 Colombian Demographic and Health Survey (ENDS in Spanish) was used as one of the main sources for comparing our national-level results with other rural contexts. As we mentioned in the introduction, perception of effective access is not commonly evaluated, and therefore, there are results from other studies with which our parameters cannot be compared, including findings related to the general population. Lastly, the results section of the article was exclusively aimed at presenting and describing the findings of our research.

We defer to the editor as to whether it is necessary to include an additional explanatory document (such as a linked file) to clarify how promotion and prevention programs and extramural activities function in Colombia, and how to compare the results in the discussion given the available sources of information. For example:

Explanation of the Functioning of Promotion and Prevention Programs in Colombia

In Colombia, promotion and prevention programs are governed and organized based on Law 1751 of 2015, of the Statutory Law for the fundamental right to health. Article 5 stipulates that the State has the responsibility to respect, protect, and guarantee the effective enjoyment of this fundamental right. Therefore, it must “Formulate and adopt policies to promote and prevent health, and to treat disease through collective and individual actions.” Thus, the Ministry of Health, through all the actors in the Colombian Social Security Health System (SGSSS, in Spanish) (Colombian Health System), must adopt and integrate approaches involving primary health care (PHC) and family and community health, and coordinate individual and collective actions according to a population focus. In order to comply with these levels of care, the Ministry issued Resolution 429 of 2019, which adopted the Comprehensive Health Care Policy (PAIS in Spanish) and the Comprehensive Health Care Model (MIAS in Spanish). One of the components of the MIAS includes comprehensive routes to health care (RIAS in Spanish), which the Colombian Health System is required to use so as to comply with mandatory health care activities.

With respect to intramural and extramural promotion and prevention activities, resolution 3280/2018 established a comprehensive route to health promotion and maintenance, a route through specific events, a route for risk groups, and a maternal-perinatal route.

These routes involve actions at the individual, collective, population, and public health management levels. They adopt technical and operational guidelines, including service providers’ compliance with procedures and specific consultations by age group, with frequencies stipulated in the technical provisions of the Resolution, for each group served. The Ministry of Health establishes indicators for evaluating and monitoring the health system, nonetheless, their construction is finalized when they are implemented in the territories. Establishing compliance parameters for the indicators depends on the diagnosis of the health situation and the territorial health plan.  Given that municipalities have different starting points, comparable standard measurements cannot be established.  Therefore, due to the differences that exist among the territories, national standards cannot be established for each program.  According to the provisions in the Colombian health system, these routes and programs must be present and operational in at least 80% of each area. (falta cita resolucion 3280/2018).

The discussion compared our results with the use of promotion and prevention programs presented in the figures, based on the available literature on population surveys in Colombia and the findings on routes from official sources. Regarding the figures that present the evaluation of our main category of analysis  —perception of access— the comparisons in the discussion were based on research studies and the available documented experience in the country and in other countries. Nevertheless, most of the available sources of information on access to health services is based on the perspective of providers rather than the users of the services. Therefore, a comparison with our results would be limited.

We do not believe that this text should or could be included in the article. It could be added as an addendum or footnote to the figures (as a linked file).

 

1. Furthermore, Figures 2 and 3 require information about the clinical specificities of the children under 1 or pregnant women to assess whether the services offered were adequate or not, perhaps according to whether their regular or schedule check-ups are in agreement with known guidelines. We are left wondering how important this information is

**RTA:**

This information cannot be recovered from our database. The data is at the household level and the question refers to someone in the household. Unfortunately, we cannot evaluate this indicator because, first, there would be very few pregnant women and children under 1 year of age. And second, we do not have a way to obtain the total children under 1 year or total pregnant women. We do know that there were few, according to the interviewers. Third, this was not the objective. Actually, the information reported does not allow for evaluating adherence to the standards in the guidelines, and that was not the study’s interest.

1. The fact that the questions ask for the last four weeks further complicates the analysis given that the person could be in a moment in which there is no need to use these services and appointments might have already happened before the four weeks period or are scheduled for after the interview.  

**RTA :**

See the complete response provided previously (comment 2, methods). An explanation about the period of 4 weeks is included. In brief, if we were to use a longer period then the time at which the program was evaluated would not be comparable for all the subjects, and would result in recall bias.

1. Figures 4 and 5 would benefit from an explanation of these programs and the guidelines or parameters established in the country regarding their offering. Are health promotion and illness prevention services replacing regular care? Why would someone have to schedule such an appointment rather than already having an appointment or being contacted to schedule the appointment? How do referrals and enrollments in these programs work? Also, questions arise about the coordination between programs and whether the presented data is adequate or follows any established guidelines. 

RTA:

See the previous response (comment 11), where towards the end we proposed including a short text about the functioning of the programs. There are no standards for the parameters that we are measuring, which we explained in the first part of the response, and it depends on the context. We are not evaluating guidelines or adherence to protocols, but rather, perception of access, subject to indicators of offerings and use of services.

We do not believe that primary care or services to prevent illness replace “regular care,” as the evaluator seems to suggest. The strategies are consistent with national public health guidelines for rural areas. We want to stress that we do not conceive of a dichotomy between health services in the context of the project and health services defined by the Public Health Plan, but rather, it is about development and adaptation in the context of the ETCRs. Regarding the last questions, we do not have an answer, we cannot provide one based on the results, and they are not part of the objective of the study. In addition, in order to answer the last three questions, information would have to be collected for that moment in time and for each ETCR, since in practice, this is different in each place and changes rapidly over time.

**Discussion:**

1. The discussion requires major changes. The authors discuss some of the results in relation to existing literature but it is unclear what are the central ideas of the discussion and what results the authors found particularly interesting and relevant.

RTA: This is a brief report of a multi-dimensional survey. That would likely occur because the objective of this first analysis is to present multi-dimensional findings in a general manner, as a basis and background for more specific analyses. It is complicated to reduce the magnitude of the different results to a few central ideas. Nevertheless, we believe the new pages, 27 and 28, contain central ideas for the discussion.

At the request of previous reviewers, the discussion is organized according to the following logic: 1. Overall context of the right to health for ex-combatants and during post-conflict, 2. Context of how this is inserted into the Colombian peace and reincorporation process, 3. Summary of the importance of this study, including strengths and weaknesses of the survey and the instrument, 4. Explanation of the main findings in the context of the ETCRs, 5. Most relevant findings, reference points, and comparison with other studies of ex-combatants, 6. Interpretation of results based on the rural context, and in contrast with other studies on effective access in rural areas, 7. Limitations of the study, and 8. Conclusions.

 

1. Page 13. 2nd paragraph. “While prioritizing the right to health for ex-combatants represents important progress in public policy, Colombia’s health system faces numerous challenges that need to be studied”. What challenges is this sentence referring to?

**RTA:**

The challenges are innumerable. Nevertheless, right after the phrase that we cited, we present the two main challenges for Colombia’s health system in terms of guaranteeing the right to health for ex-combatants. One challenge is rural health, which is explained by the fact that ETCRs are located in rural areas and there is little guarantee of access to health services in those areas in Colombia. The second challenge that we expressed is that by not fulfilling the guarantee of access to health services for ex-combatants, which is one of the cornerstones of compliance with reintegration and reincorporation, the peace process itself could be at risk.

1. Page 13. 3rd paragraph. The ARN survey of 2014 describes a different reintegration program. This program includes ex-combatants who demobilized before the peace agreement and the majority of them moved to urban areas. As mentioned, the article should specify from the beginning that the population assessed in the study are part of the collective reincorporation program and describe their main characteristics. Is it possible to discuss some differences between access to health care depending on whether the ex-combatants participated in the reintegration or the reincorporation programs?

**RTA:**

Some of these points have already been addressed in the new version of the introduction, which provides greater context. It is now clear that we refer to reincorporation. Throughout the introduction we now refer to reincorporation and how it is inserted into the Health for Peace project. We corrected where the term “reintegration” was incorrectly used, where it was necessary to replace it with reincorporation. The terminology for discussing reincorporation was verified and is now consistent with the program, IOM, and the institutions.

Nevertheless, we are not able to address the last question based on the results that are available to us, since we would not be able to make distinctions based on the information that was collected.

Even if differences between access to health services by the different reintegration and reincorporation processes could be compared, it would not fall within the scope of this study since the objective proposed for this work was to determine the perception of effective access to health services according to the activities provided by the project. This is a cross-sectional  measurement that is not comparable with other reintegration and reincorporation processes developed in the country, for two fundamental reasons. One reason is because it is organized by official entities that do not have public information about access to health services according to reintegration and reincorporation.  Secondly, these programs measure access based only on enrollment in the health system. Furthermore, this research did not differentiate between whether they belonged to one program or the other.

 

1. The contrast with healthcare provision in other rural areas is an interesting and important discussion. However, the main points of this contrast are not clear. 

**RTA Oficial **:

In paragraphs 14, 15, and 16 in the discussion, we contrasted the provision of services with other rural areas based on the available literature, which indicated that their availability is very limited. The only source for comparing access to services in rural areas in Colombia is the 2015 National Demographics and Health Survey, which contains some sections that distinguish between type of enrollment and type of services for the rural population.  Nevertheless, as can be seen, that survey does not involve perception of access, which is the category analyzed in our study. This greatly limits our ability to compare our results on access in rural areas with those of ENSUT, since the ENSUT results were oriented more towards service provides than users.

In general, evaluations of access to services in Colombia are more focused on urban areas, such as the Avillarraga et al survey, and there is still a large gap in knowledge about what occurs in rural areas. Therefore, our results provide an initial basis for this line of study, and contribute to reducing the knowledge gap.

Furthermore, in order to discuss our findings in greater depth, we used other international sources, such as the studies obtained from Paraguay and some areas of Africa, where the study of access to services in rural areas is a little more common. But we reiterate that there is a limitation given the type of construct that is used to evaluate access.

Therefore, we accept your comments that perhaps our analysis of the subject does not have clear points of contrast. Nonetheless, this is related more to the existing literature and knowledge on the subject, thereby demonstrating the importance of continuing to more deeply explore this subject and generate more knowledge about it.

.

1. Page 14 3rd paragraph. We suggest avoiding adjectives such as “high”, “low” and “small” without providing specific data.

RTA:  These changes were made. The adjectives were replaced with specific data from the surveys.

1. The discussion of the results about health promotion and illness prevention services in relation to studies conducted in Manizales and Medellin does not seem appropriate, unless the difference between these services in rural and urban areas is stated.  

RTA: This is true, but since the most relevant points of reference do not exist, we prefer to keep this in the text. Although the reviewer is clearly correct and a clarification for the reader is needed.

We clarified this in the paragraph before the discussion about the studies. Studies of urban areas were used because we did not find any studies of promotion and prevention services in rural areas in Colombia that presented findings similar to ours, although their correspondence to different socioeconomic and cultural contexts should certainly be taken into account.

1. What do the authors mean by good treatment in accordance with their culture and beliefs? Are the notions of culture and beliefs related to their identity as ex-combatant, a regional identity, a rural identity, or an ethnic/racial identity? 

**RTA**:

The dimension of treatment according to culture and beliefs was included in the instrument for two reasons: providing culturally appropriate health care for the ex-combatant population is one of the cornerstones of the Health for Peace project, as reflected in the explanation of the components of the project. And, as mentioned in the introduction to the article, one of the dimensions underlying the perception of effective access to health services is that the care provided by health personnel is acceptable to the users. This category does not reflect a specific belief system or culture, but rather, it ensures that when health personnel provide health care to the ex-combatant population and their family, they take into account the user’s opinion, acceptance, and wishes regarding activities and treatments for managing and treating their illnesses and caring for their health.

1. The results do not indicate that people’s behavior to seek health care services can be explained through the health belief model.

RTA :

This is relevant given how it is presented in the article, and should be kept in the discussion.

One of the dimensions of effect access to health services is self-agency, which does not depend on the health system but rather on people’s attitudes and beliefs about seeking health services in a timely fashion. Our results found that 41% of the population knew about the extramural services offered by the project but only 17% used them. It is clear that this was not due to a lack of knowledge about the existence of the service, but rather, to the individual’s own interest in using the services.

This belief system is of course a construct that goes beyond the dimension of self-agency in access to health services, and is complemented by bio-psycho-social models that more thoroughly explore the components of a person's belief system, such as the health beliefs model, which can give a more explanatory framework for specific findings related with extramural services. In regard to these services, in addition to knowing about their existence, people need to be motivated to use them, and  is determined by the priority that each individual places on health, on each one of its dimensions, as well as by self-agency and self-care.

In the discussion, the authors would like to offer possible explanations for our results without establishing a definitive position, but rather, by offering possible explanations that are supported by a rigorous literature search on the subject.

 

1. . We suggest that the center of the discussion should be around how the current data compare to the challenges described for other rural populations. The authors hint in this direction but could make significant contributions by signaling how ex-combatants’ perception of their access to health care compares to other studies in rural areas. Are they getting better or worse access to health care than other rural communities? What does this say about the reincorporation process and about the health care system? If the analysis is broken down by region, what does it tell us about different regions in Colombia and, perhaps, different “reincorporation processes”? 

**RTA:  **The questions by the reviewers go well beyond the scope of our results. Our interest is to merely describe a snapshot in time of the process in the ETCRs. We provide context and reference points, but in order to answer the questions that are posed, specifically if it has improved or worsened over time, would require other types of data (probably longitudinal), different sources of primary and secondary information,  and different and more complex methodologies, all of which goes beyond the scope of our results.

We do not agree with discussing aspects that can not directly enter into dialogue with the results of our study, given that this is a report of a descriptive study. This analysis would warrant another investigation, and we prefer to be cautious since we do not have elements with which to even suggest a response.

As we explained, we presented the data by region for informative purposes, and given our sample size and design, regions cannot be compared. Therefore, it would not be possible to explore any comparisons among regions with our results.

We limit ourselves to highlighting and comparing only the results from other studies that can enter into dialogue with our results and that pertain to our objective.

The main objective of the research presented in this article was to determine the perception of effective access to health services by a population of ex-combatants who were in the process of reincorporation and reintegration in the ETCRs.

In this case, the Health for Peace project sought to strengthen the Colombian health system through joint actions, thereby facilitating and enhancing access to health services for this population and their families, in the area of primary health care, since the ETCRs are located in rural environments where access to these types of services presents difficulties, and there are significant gaps for the rural population in general.

An instrument was designed to determine effective access to health services in order to identify whether these types of actions were strengthening the services and thereby impacting the health of the ex-combatant population while the project’s actions were underway. The instrument was designed for this population from the perspective of the user. That is, it was based on the construct of perception, which more adequately indicates whether the project strengthened the health system such that ex-combatants and their families had the impression or appreciation that they were effectively accessing the services that they needed —whether faced with an emergency or when seeking health promotion and prevention services through intramural and extramural activities.

With regard to the regional comparability of the results, as explained in the discussion, one of the few available sources for making a comparison is a study by the World Bank, which is the only source that was found that evaluates these dimensions by region in Colombia. That is, the regions with the least access are Orinoquia and Amazonia, which is due to the little availability of health centers, whereas central Colombia is characterized by a greater presence of this type of health institution.

With regard to comparing different reincorporation processes, the contrast at the global level was included at the beginning of the discussion, in paragraphs 2 to 4. Here, Colombia was described as representing a high standard, as the health component is one of its priorities, whereas other countries, such as Namibia, did not take this component into account or it only involved the supply of health kits for ex-combatants.

With regard to the experience with reincorporation processes in other countries, the health dimension has not been prioritized or mentioned in the available sources. Thus, it is important to document Colombia’s experience with the reincorporation route, which we attempted to point out in the beginning of the discussion, in paragraphs 4 and 5.

In addition. the data on reincorporation processes and routes that are available from official sources measure access to services based only on enrollment in the Colombian health system and frequency of use of services. Considering the scope of our results, that is not comparable. Based on our findings, we can conclude that the population had a favorable perception of the actions that were taken to strengthen the Colombian health system in rural areas, such as those by the project. We also conclude that compliance with reincorporation processes and their successful completion require actions that ensure that the health dimensions continue to be priorities, but this must be based on the rural context, which is a great challenge for the health system.
